# Lactobacillus Dominate in the Intestine of Atlantic Salmon Fed Dietary Probiotics

**DOI:** 10.3389/fmicb.2018.03247

**Published:** 2019-01-11

**Authors:** Shruti Gupta, Adriána Fečkaninová, Jep Lokesh, Jana Koščová, Mette Sørensen, Jorge Fernandes, Viswanath Kiron

**Affiliations:** ^1^Faculty of Biosciences and Aquaculture, Nord University, Bodø, Norway; ^2^Department of Food Hygiene and Technology, University of Veterinary Medicine and Pharmacy in Košice, Košice, Slovakia; ^3^Department of Microbiology and Immunology, The University of Veterinary Medicine and Pharmacy in Košice, Košice, Slovakia

**Keywords:** fish, *Salmo salar*, feed additive, probiotics, intestinal bacteria, *Lactobacillus*, microbiota, amplicon sequencing

## Abstract

Probiotics, the live microbial strains incorporated as dietary supplements, are known to provide health benefits to the host. These live microbes manipulate the gut microbial community by suppressing the growth of certain intestinal microbes while enhancing the establishment of some others. Lactic acid bacteria (LAB) have been widely studied as probiotics; in this study we have elucidated the effects of two fish-derived LAB types (RII and RIII) on the distal intestinal microbial communities of Atlantic salmon (*Salmo salar*). We employed high-throughput 16S rRNA gene amplicon sequencing to investigate the bacterial communities in the distal intestinal content and mucus of Atlantic salmon fed diets coated with the LABs or that did not have microbes included in it. Our results show that the supplementation of the microbes shifts the intestinal microbial profile differentially. LAB supplementation did not cause any significant alterations in the alpha diversity of the intestinal content bacteria but RIII feeding increased the bacterial diversity in the intestinal mucus of the fish. Beta diversity analysis revealed significant differences between the bacterial compositions of the control and LAB-fed groups. *Lactobacillus* was the dominant genus in LAB-fed fish. A few members of the phyla Tenericutes, Proteobacteria, Actinobacteria, and Spirochaetes were also found to be abundant in the LAB-fed groups. Furthermore, the bacterial association network analysis showed that the co-occurrence pattern of bacteria of the three study groups were different. Dietary probiotics can modulate the composition and interaction of the intestinal microbiota of Atlantic salmon.

## Introduction

The ecological community of microorganisms that reside (Marchesi and Ravel, 2015) in the gastrointestinal tract (GIT) of an organism is referred to as the gut microbiota (Lozupone et al., 2012). The GIT of a healthy human harbors a dense (Kelsen and Wu, 2012; Marchesi et al., 2016) and diverse population (Lozupone et al., 2012) of commensal microorganisms, which offer many benefits to the host, including immune homeostasis and health maintenance (Sommer and Bäckhed, 2013). These commensal gut bacteria are also known to aid in amino-acid production (Lin et al., 2017), nutrient metabolism and absorption (Morowitz et al., 2011; Semova et al., 2012), vitamin and bioactive metabolite' synthesis (Cummings and Macfarlane, 1997; LeBlanc et al., 2013), and pathogen displacement (Kamada et al., 2013). An imbalance in the gastrointestinal microbial composition can lead to immune-mediated diseases (Petersen and Round, 2014). A healthy gut bacterial assembly is essential for the well-being of the host organisms including fish, the microbiome of which is shaped by environment- and host-related factors (Wong and Rawls, 2012; Eichmiller et al., 2016; Lokesh et al., 2018).

Probiotics are “living bacteria,” and when they are administered as supplements in the right amount they can confer health benefits to humans (FAO and WHO, 2006), by targeting, among others intestinal health through stimulation of intestinal epithelial cell proliferation and differentiation, fortification of intestinal barrier and immunomodulation (Gareau et al., 2010; Thomas and Versalovic, 2010; Hemarajata and Versalovic, 2013). Probiotics also have both direct and indirect effects on the intestinal microbial composition and diversity, and global host metabolic functions (Scott et al., 2015). These live bacteria produce antimicrobial compounds that suppress the growth of other microorganisms and compete for their receptors and binding sites (Spinler et al., 2008; O'Shea et al., 2012); thus altering the gut microbiota (Collado et al., 2007). Members of the genera *Lactobacillus* and *Bifidobacterium* are the most commonly used probiotic organisms for humans (O'Toole and Cooney, 2008).

Lactic acid bacteria (LAB) maintain intestinal health by producing lactic acid that can be utilized by short-chain fatty acids (SCFAs)-producing microorganisms. SCFAs (particularly acetate, propionate and butyrate) contribute to host health maintenance; for example, butyrate is used as energy source by the intestinal epithelial cells and also have anti-inflammatory effects on the host cells (Louis et al., 2014). LAB that is generally found in the GIT of endothermic animals have been extensively investigated and their benefits have been reviewed by many researchers (Pavan et al., 2003; Masood et al., 2011; Yang et al., 2015; Karamese et al., 2016). The importance of fish gut-dwelling LAB in aquaculture has been described in other reviews (Ringø and Gatesoupe, 1998; Gatesoupe, 2008). *Lactobacillus* that colonize the intestinal regions of fish are able to evoke immune responses and impart protection against diseases (He et al., 2017).

Feeding diets supplemented with beneficial bacteria such as LAB is being considered as an alternative approach to control diseases in farmed fish (Martínez Cruz et al., 2012; Fečkaninová et al., 2017; Rodriguez-Nogales et al., 2017). Not many studies in fish have employed high-throughput sequencing techniques to understand the changes in bacterial communities following LAB feeding. In this study, we examined the ability of *Lactobacillus* to modulate the distal intestinal microbiota of Atlantic salmon, a farmed salmonid fish. In addition, we describe the differences in the topology of co-occurrence networks associated with the intestinal bacteria of Atlantic salmon offered feeds with and without *Lactobacillus*.

## Materials and Methods

### Ethics Statements

This study was approved by the Norwegian Animal Research Authority, FDU (Forsøksdyrutvalget ID-7898). Fish handling and sampling procedures were in compliance with the description in LOVDATA. The rearing water was treated with UV rays to remove substances that could be harmful to the fish. Optimum values for water salinity, oxygen and nitrogen concentration were maintained in the rearing tanks. The temperature of the fish rearing hall was kept stable during the entire feeding experiment.

### Test Probiotics, Feed Type, and Design

Two species of *Lactobacillus* (RII and RIII) that were previously isolated from the intestinal content of farmed healthy juveniles of rainbow trout (commercial fish farm–Rybárstvo PoŽehy s.r.o., Slovak Republic) were employed in this study. Antimicrobial susceptibility of the microorganisms was assessed based on the “Guidance on the assessment of bacterial susceptibility to antimicrobials of human and veterinary importance” provided by the European Food Safety Authority. Sensitivity or intrinsic resistance of the isolated organisms to a recommended set of antibiotics make them safe for use as probiotics in aquaculture. Both RII and RIII showed antagonistic activity against salmonid pathogens *Aeromonas salmonicida* subsp. *salmonicida* CCM 1307 and *Yersinia ruckeri* CCM 6093 (Fečkaninová, 2017). Furthermore, high level of tolerance to different pH, bile, temperature, and high growth properties of the two species were confirmed through *in vitro* studies (Fečkaninová, 2017). The test probiotics were coated on commercial salmon feeds. Briefly, a pure culture of probiotic bacteria that were grown on de Man, Rogosa and Sharpe agar (MRS) plates (HiMedia, Mumbai, India) for 48 h were inoculated into 1,000 ml of MRS broth and incubated for 18 h at 37°C. The culture was centrifuged at 4,500 rpm for 20 min at 4°C in a cooling centrifuge (Universal 320 R, Hettich, Germany). The resulting cell pellets were washed twice and resuspended in 30 ml of 0.9% (w/v) sterile saline. The feed (batches of 1,800 g) was thoroughly coated with the bacterial suspensions (Spirit Supreme, Skretting AS, Norway) using a vacuum coater (Rotating Vacuum Coater F-6-RVC, Forberg International AS, Norway). The bacterial counts on feeds were ~10^8^ cells.g^−1^ (RII/RIII), as determined by spread plating on MRS agar plates and incubating for 48 h at 37°C. The control feeds were coated with 0.9% of sterile saline alone. The coated feeds were stored at 4°C until they were offered to Atlantic salmon.

### Experimental Fish, Feeding Regime, and Environmental Parameters

Atlantic salmon of average weight 522 ± 68 g were maintained in 800 L tanks in a flow-through seawater system, earlier described in Sørensen et al. (2017). A 20-day feeding trial was conducted at the research station, Nord University, Bodø, Norway. Three groups of fish (*n* = 45 fish/tank; 3 replicate tanks per group) were offered feeds with (RII ~10^8^ cells.g^−1^-RII; RIII ~10^8^ cells.g^−1^-RIII) or without probiotics (Control—C). The fish were fed *ad libitum*; the feeds were dispensed two times a day, between 08.00–09.00 and 14.00–15.00, using automatic feeders (Arvo-Teck, Huutokoski, Finland). The water flow rate, temperature, salinity and O_2_ levels in the tanks were 800 L/h, 6.7–7.1°C, 33 ppt, >85% saturation measured at the outlet, respectively. A photoperiod of 24:0 LD was maintained throughout the feeding trial.

### Collection of the Intestinal, Tank Biofilm, and Rearing Water Samples

First, the fish were euthanized using 160 mg/L of MS222 tricaine methanesulfonate (Argent Chemical Laboratories, Redmond, WA, USA). Thereafter, the body surface of the fish was swiped with 70% ethanol. The fish were then dissected to aseptically remove the GIT from the abdominal cavity. The distal intestinal (DI) region was separated from the GIT and the content and surface mucus samples from the DI were collected (*n* = 18 for each group; 6 fish/tank) using sterile forceps and sterile glass slides, respectively. In addition to these fish samples, we collected environmental samples: water from the main inlet to the rearing hall (inlet water, *n* = 1), water from the rearing tanks (*n* = 3) and biofilm from the walls of the rearing tanks (*n* = 3). From the 3 tanks of each group, one liter of rearing water was filtered using 0.2 μm pore-size filters (Pall Corporation, Hampshire, United Kingdom) and the filter paper was stored at −80°C. The biofilm samples were scraped from the walls of the 3 tanks of each group. The fish and biofilm samples were collected in cryotubes, snap-frozen in liquid nitrogen and stored at −80°C.

The sample abbreviations reported in this article are: (i) fish samples–Control distal intestine content (CDC), RII distal intestine content (RIIDC), RIII distal intestine content (RIIIDC), Control distal intestine mucus (CDM), RII distal intestine mucus (RIIDM), RIII distal intestine mucus (RIIIDM); (ii) environmental samples– Control tank water (CW), RII tank water (RIIW), RIII tank water (RIIIW), inlet water (IW), Control tank biofilm (CB), RII tank biofilm (RIIB), RIII tank biofilm (RIIIB).

### DNA Extraction and PCR Amplification of Bacterial 16S rRNA Gene for Illumina MiSeq Amplicon Sequencing

Genomic DNA was extracted from the content, mucus and biofilm samples using the Quick-DNA™ Fecal/Soil Microbe 96 kit (Zymo Research, Irvine, CA, USA) following the manufacturer's protocol. Metagenomic DNA Isolation kit for water (Epicenter Biotechnologies, Madison, WI, USA) was employed to extract the genomic DNA from the water samples. The quality of the extracted DNA was checked on 1.2% (w/v) agarose gel. Qubit 3.0 fluorometer (Life Technologies, Carlsbad, USA) was employed to quantify the concentration of DNA.

To describe the changes in the intestinal bacteria under the influence of LAB, we amplified the V3–V4 region of the bacterial 16S rRNA gene employing a dual-index sequencing strategy described by Kozich et al. (2013). The PCR reactions were carried out in triplicates, each reaction (25 μl) volume contained 12.5 μl of Kapa HiFi Hot Start PCR Ready Mix (KAPA Biosystems, Woburn, USA), 1.5 μl of each forward and reverse primer (at a final concentration of 100 nM), 3.5 μl of DNAse and nuclease free water (Merck, Darmstadt, Germany) and 6 μl of DNA template and/ or 6 μl of negative PCR control. The thermocycling conditions included initial denaturation at 95°C for 5 min, followed by 35 cycles of denaturation at 98°C for 30 s, annealing at 58°C for 30 s, extension at 72°C for 45 s, and the final extension performed at 72°C for 2 min. After performing the PCR, the resulting amplicon triplicates were pooled and visualized on 1.2% (w/v) agarose gel stained with SYBR^®;^ Safe (Thermo Fisher Scientific, Rockford IL, USA), and the amplicon size was compared to a 1 kb DNA ladder (Thermo Fisher Scientific, Inc.). No amplification was observed in the negative PCR control. Only the amplicons (~550 bp) with clear visible bands were selected, purified using the ZR-96 Zymoclean™ Gel DNA Recovery Kit (Zymo Research) and eluted in 15 μl of elution buffer. The eluted amplicon library (sequencing library) was quantified by qPCR using the KAPA Library Quantification Kit (KAPA Biosystems). After quantification, each amplicon library was normalized to an equimolar concentration (3 nM) and validated on the TapeStation (Agilent Biosystems, Santa Clara, USA), prior to sequencing. The normalized library pool was further diluted to 12 pM, spiked with equimolar 10% Phix control and then paired-end sequencing was performed using the 600 cycle v3 sequencing kit on the Illumina MiSeq Desktop sequencer (Illumina, San Diego, CA, United States) in 2 runs with inter-run calibrators to reduce eventual differences between sequencing runs.

### 16S rRNA Gene Sequence Data Processing

*Sequence data quality check, processing and analyses:* The sequence quality of the raw reads generated from the Illumina MiSeq machine was checked using FastQC (Andrews, 2010). The forward reads (R1) corresponding to V3 region were employed for subsequent analyses because they were of better quality than the reverse reads (R2) corresponding to V4 region [Phred quality score (Q) ≤ 15]. Sequence processing was performed using the UPARSE (USEARCH version 9.2.64) software by Edgar (2013); this step included quality filtering and operational taxonomic units OTU clustering. FastQ files were used as the input file for the UPARSE pipeline. The raw reads were truncated to 240 bp and quality-filtered. The reads were truncated to remove the low-quality base pairs at the 3′-end and to make all samples of same sequence length. Furthermore, chimeric sequences were removed using the UCHIME algorithm (Edgar et al., 2011). The quality-filtered sequences were clustered into OTUs at 97% sequence similarity level. For taxonomy prediction, we employed the 16S rRNA Ribosomal Database Project (RDP) training set with species names v16. This RDP training set was used as a reference database because the large 16S databases like SILVA, Greengenes, or the full RDP database may give unreliable annotations of short 16S rRNA tags (Edgar, 2018). Taxonomic ranks were assigned to the OTUs using the SINTAX algorithm (Edgar, 2016) using a bootstrap cutoff value of 0.5. Afterwards, OTUs with a confidence score < 1 at the domain level and the OTUs belonging to the phyla Cyanobacteria and Chlorophyta were removed to exclude the plant-related sequences from the microbiota analysis. After constructing the OTU table, the counts were rarefied to the lowest number of sequences per sample to get an even sampling depth to facilitate comparisons between the treatment groups. The OTU count data was divided into 4 sets based on the sample type, namely the DI content, DI mucus, tank water and tank biofilm samples. The downstream analyses were performed separately on these 4 sets. Furthermore, to ensure that we employ content and mucus data from the same fish, only 14 fish from each group were considered for the downstream analyses. In total 103 samples were used for the downstream analyses, including the tank water and biofilm samples. The raw 16S rRNA gene sequence data from this study has been deposited in the European Nucleotide Archive (ENA) under the accession number ERP110004.

*Analyses of microbial diversity and composition:* R codes were executed in RStudio v3.5.0 (RStudio Team, 2016) and the functions of the R packages “iNEXT” v2.0.12 (Hsieh et al., 2016), “phyloseq” v1.22.3 (McMurdie and Holmes, 2013) and “ggplot2” v2.2.1 (Wickham, 2016) were used to make the rarefaction curves for the species richness, to calculate and visualize diversity indices, and to prepare the abundance plots. Another R package called “microbiome” v1.0.2 (Lahti et al., 2017) was used to make core and rare microbiota (relative abundance of core taxa) plots. Alpha diversities were calculated based on the formula suggested by Jost (2006); for Shannon diversity (effective number of common OTUs) and Simpson diversity (effective number of most abundant OTUs). Beta diversity was examined by conducting weighted UniFrac distance metric (for fish samples)-based PCoA and double principal coordinates analysis (DPCoA, for water and biofilm samples) (Fukuyama et al., 2012).

**Figure 1:**
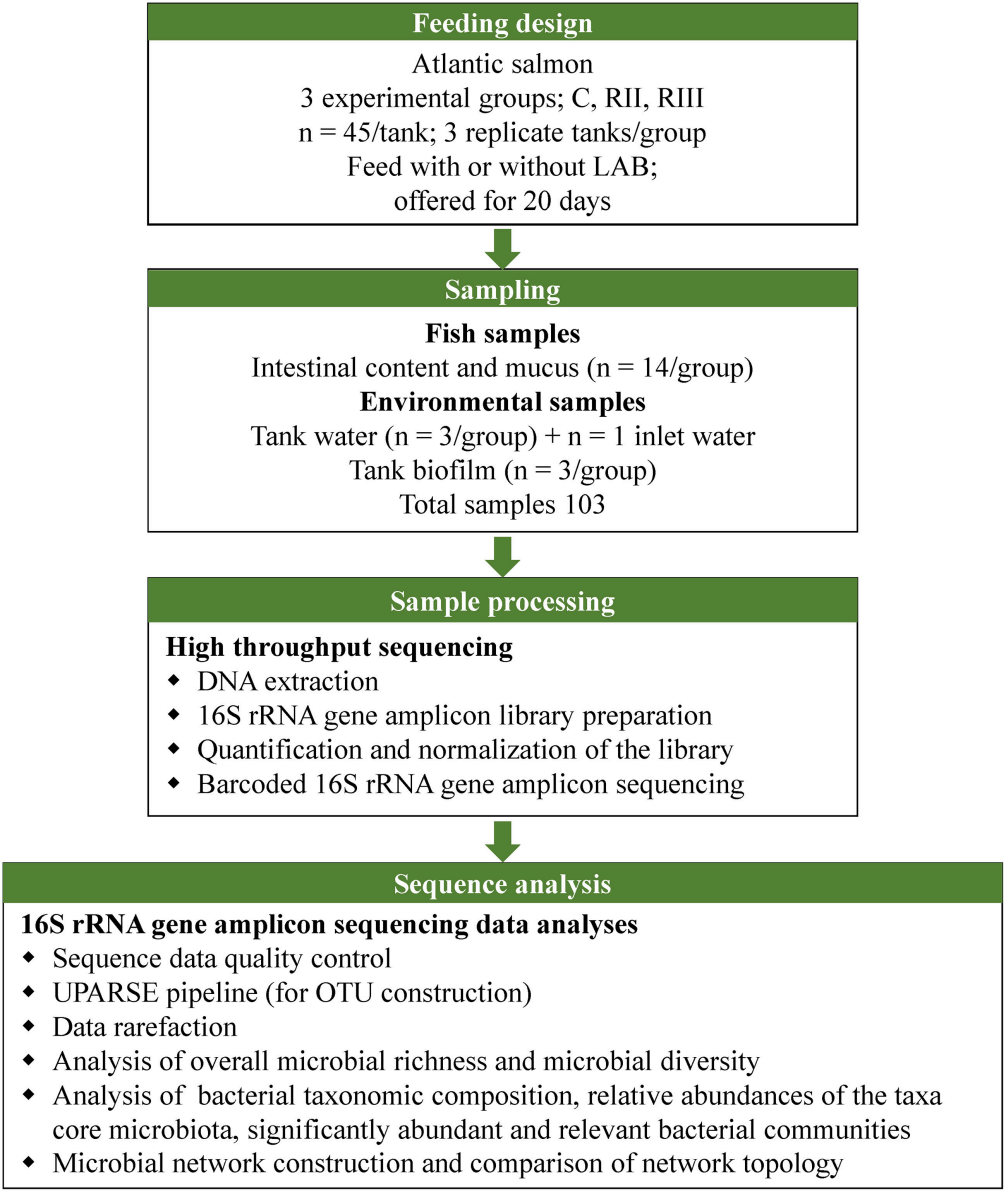
Illustration of the study design, sampling and sequence analyses.

The feeding design, sample processing and sequencing, and analyses are shown in Figure 1.

### Statistical Analysis of the Bacterial 16S rRNA Gene Amplicon Data

Statistical analysis was also performed in RStudio v3.5.0. Kruskal-Wallis test followed by Dunn's test was employed to detect differences in alpha diversity, and we report statistically significant differences at *p* < 0.05 and statistical trends at *p* ≤ 0.15. Betadisper was used to check the assumption of heterogeneity in dispersions; after that Adonis (PERMANOVA) followed by pairwise comparisons was employed (999 permutations) to understand the significant dissimilarities of the communities. “ANCOM” v1.1–3 (Mandal et al., 2015) was used to detect the differentially abundant OTUs in the treatment groups, and “Boruta” v5.3.0 R package (Kursa and Rudnicki, 2010) was employed to find the relevant OTUs that caused the differences in the intestinal bacteria of the three fish groups.

### Microbial Network Construction and Comparison of Topology

We used “SPIEC-EASI” v0.1.4 R package (SParse InversE Covariance Estimation for Ecological Association Inference) for generating the single-domain bacterial network. SPIEC-EASI is a statistical method that assumes the underlying microbial interaction networks to be sparse (Kurtz et al., 2015). In this study, we employed the neighborhood selection (MB) method on the sequenced 16S rRNA gene (V3 region) data of both DI content and mucus samples to understand the community organization.

We explored the co-occurrence networks to uncover the probable biological interactions occurring within the microbial communities. We used the top 200 OTUs for network construction, since it is advised to avoid extremely rare OTUs or OTUs with a large number of zeros (Banerjee et al., 2018). The co-occurrence microbial networks were constructed and analyzed using the functions of the R package “igraph” v1.2.1 and customized ggplot2 commands. A network consists of a set of vertices (commonly called as nodes) and set of edges. The degree of a node is the number of connections it has with the other nodes in the network. Betweenness estimates the number of shortest paths that pass through the nodes in the network and assortativity coefficient quantifies the extent of the selectively connected labeled pair of nodes (Kolaczyk and Gábor, 2014). We compared the topology of the networks of the content and mucus samples separately by analyzing the node degrees and betweenness of the control and LAB-fed groups using Kruskal-Wallis test followed by Dunn's test.

## Results

We analyzed the V3 region amplicons of the 16S rRNA gene that was sequenced on our high-throughput sequencing platform. A total of 28,747,884 high-quality reads were clustered into 1,823 OTUs at 97% identity threshold. These reads were rarified based on sample-size to 12,855 reads/sample; this allowed us to assess most of the underlying microbial diversity (Supplementary Figures 1A,B).

The differences in the DI bacterial communities of the LAB-fed fish compared to the control fish are explained based on the following diversity metrics: overall microbial richness (i.e., counts of individual OTUs), effective number of OTUs (counts of common and dominant OTUs), taxonomic composition, relative abundances of the bacterial taxa. Furthermore, we present the significant and relevant bacterial communities of the DI microbiota. We also describe the topology of the networks of the bacterial communities in the 3 fish groups.

### Differences in the Microbial Diversity and Composition of the Intestinal and Environmental Microbiota

**Figure 2:**
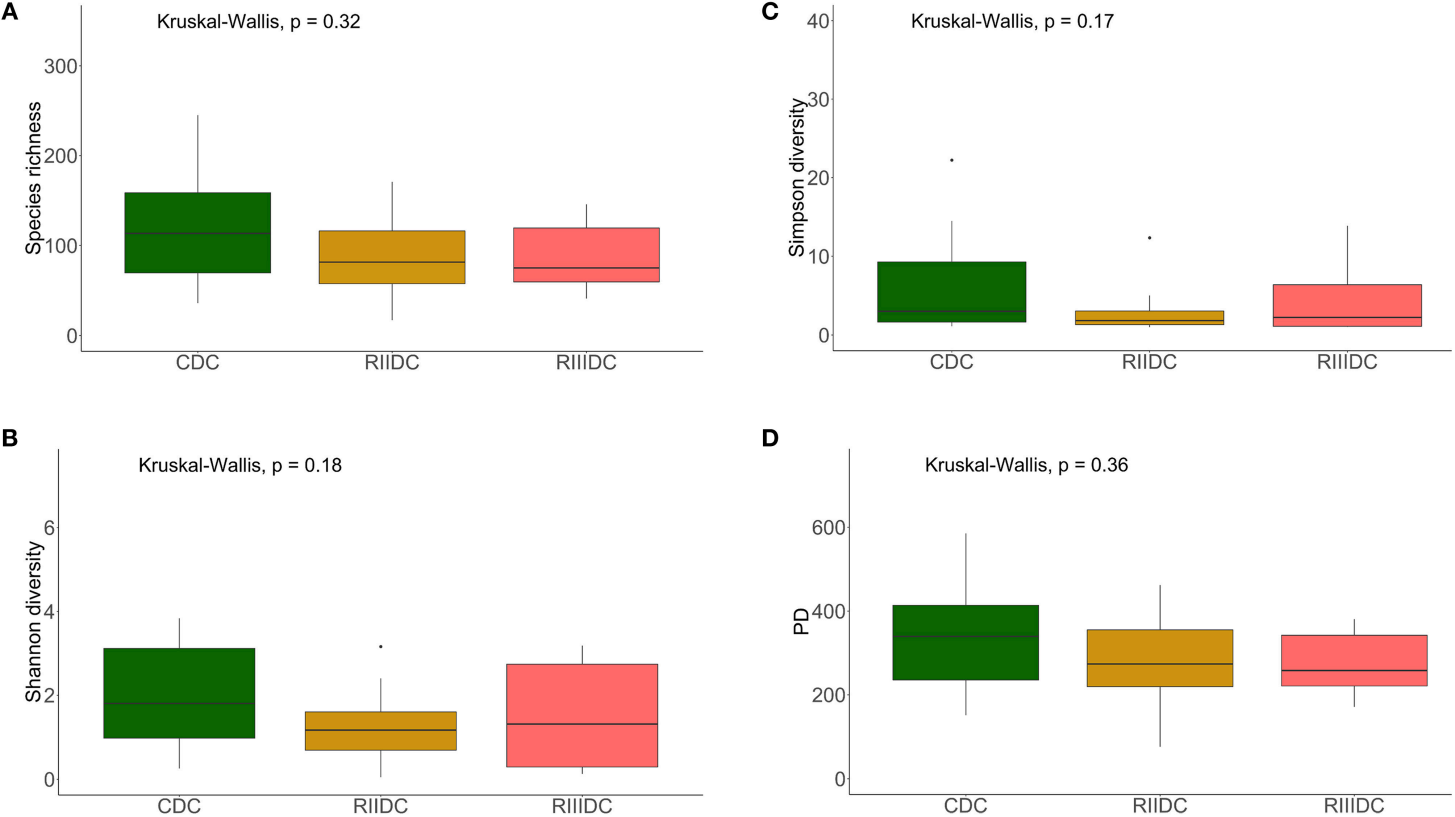
Diversity of the bacterial communities of the intestinal content. Boxplots show the species richness **(A)**, Shannon index **(B)**, Simpson index **(C)**, and Faith's phylogenetic diversity **(D)**. The feed group codes are as follows: Control, CDC; RII, RIIDC; RIII, RIIIDC.

**Figure 3:**
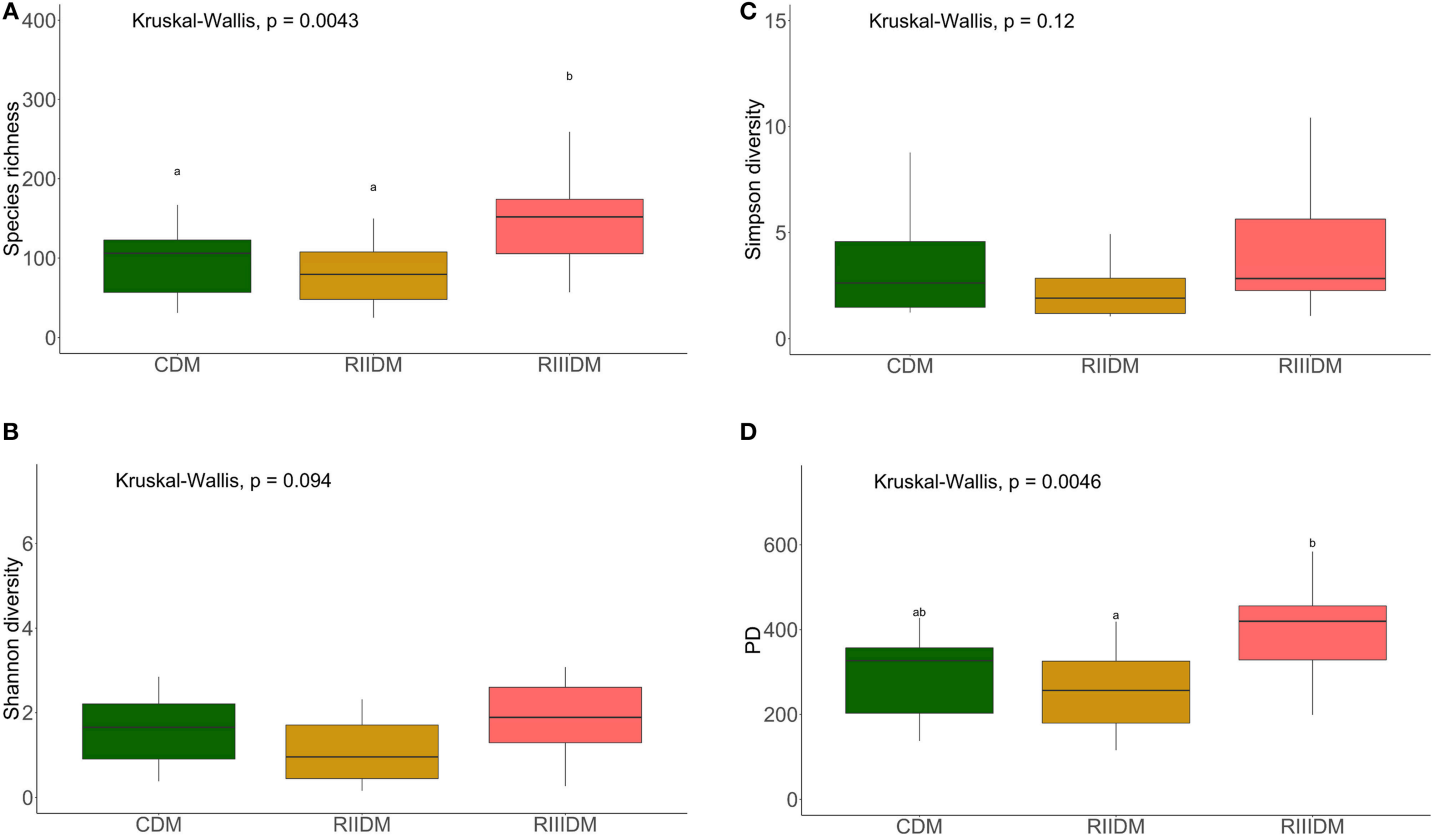
Diversity of the bacterial communities of the intestinal mucus. Boxplots show the species richness **(A)**, Shannon index **(B)**, Simpson index, **(C)** and Faith's phylogenetic diversity **(D)**. Different letters indicate statistically significant differences (*P* < 0.05) between the study groups. The feed group codes are as follows: Control, CDM; RII, RIIDM; RIII, RIIIDM.

**Figure 4:**
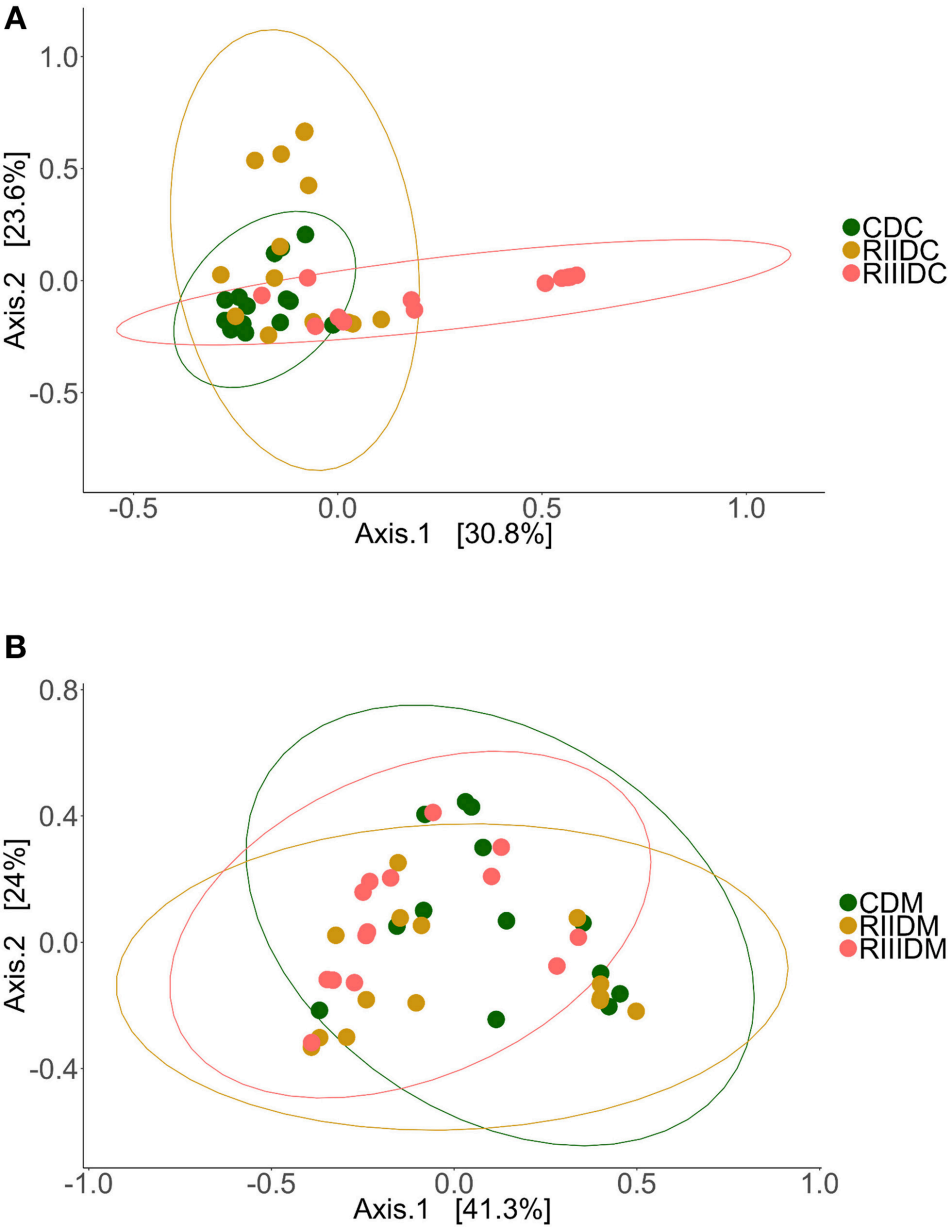
Beta diversity of the intestinal bacterial communities. Principal coordinate analysis plot **(A)** shows the differences in the composition of the bacterial communities in the intestinal content (Control, CDC; RII, RIIDC; RIII, RIIIDC). Principal coordinate analysis plot **(B)** shows the differences in the composition of the bacterial communities in the intestinal mucus (Control, CDM; RII, RIIDM; RIII, RIIIDM).

LAB feeding did not affect the species richness of the bacterial community in the DI content (Figure 2A). However, this was not the case for bacteria in the DI mucus; the species richness was found to be higher in the mucus of the RIII-fed group (*p* = 0.004 for RII vs. RIII and *p* = 0.071 for RIII vs. C) (Figure 3A). We observed differences in the effective number of common and dominant OTUs in the mucus of LAB-fed groups, (*p* = 0.109 and *p* = 0.146 for RII vs. RIII; Figures 2B, 3B and Figures 2C, 3C). Comparison of the Faith's phylogenetic diversity (PD) of the DI content did not reveal any significant differences (Figure 2D). For the DI mucus, differences were observed between the PD associated with the three fish groups (*p* = 0.004 for RII vs. RIII and *p* = 0.079 for RIII vs. C; Figure 3D). It is noteworthy that the median alpha diversities of RII lies below the corresponding values of C although we did not detect a trend or statistically significant difference between the feed groups. PCoA based on the weighted UniFrac distance matrix revealed the beta diversity of the bacterial communities; the differences between the control and LAB-fed groups were statistically significant (Figure 4A: F statistic = 9.215, *R*^2^ = 0.320, *p* < 0.001; and Figure 4B: F statistic = 3.114, *R*^2^ = 0.137, *p* < 0.002).

The beta diversity of the bacterial communities in the rearing tank water and biofilm samples were also analyzed. The bacterial communities in the water of the 3 study groups were not different (Supplementary Figure 2A, F-statistic = 0.753, *R*^2^ = 0.273, *p* = 0.684), as was the case with the bacteria in the biofilm (Supplementary Figure 3A, F statistic = 0.681, *R*^2^ = 0.185, *p* = 0.574). On the other hand, the bacterial communities in the water were significantly different from those of the fish (Supplementary Figures 2B–G). Although we did not observe any significant differences between the bacterial communities of the tank biofilm and the intestinal mucus bacteria of the LAB-fed fish (Supplementary Figures 3B–D,F–G), the biofilm and mucus bacteria of the control group were different (Supplementary Figure 3E, F statistic = 16.29, *R*^2^ = 0.520, *p* = 0.003).

### Intestinal Bacterial Composition Under the Influence of LAB

**Figure 5:**
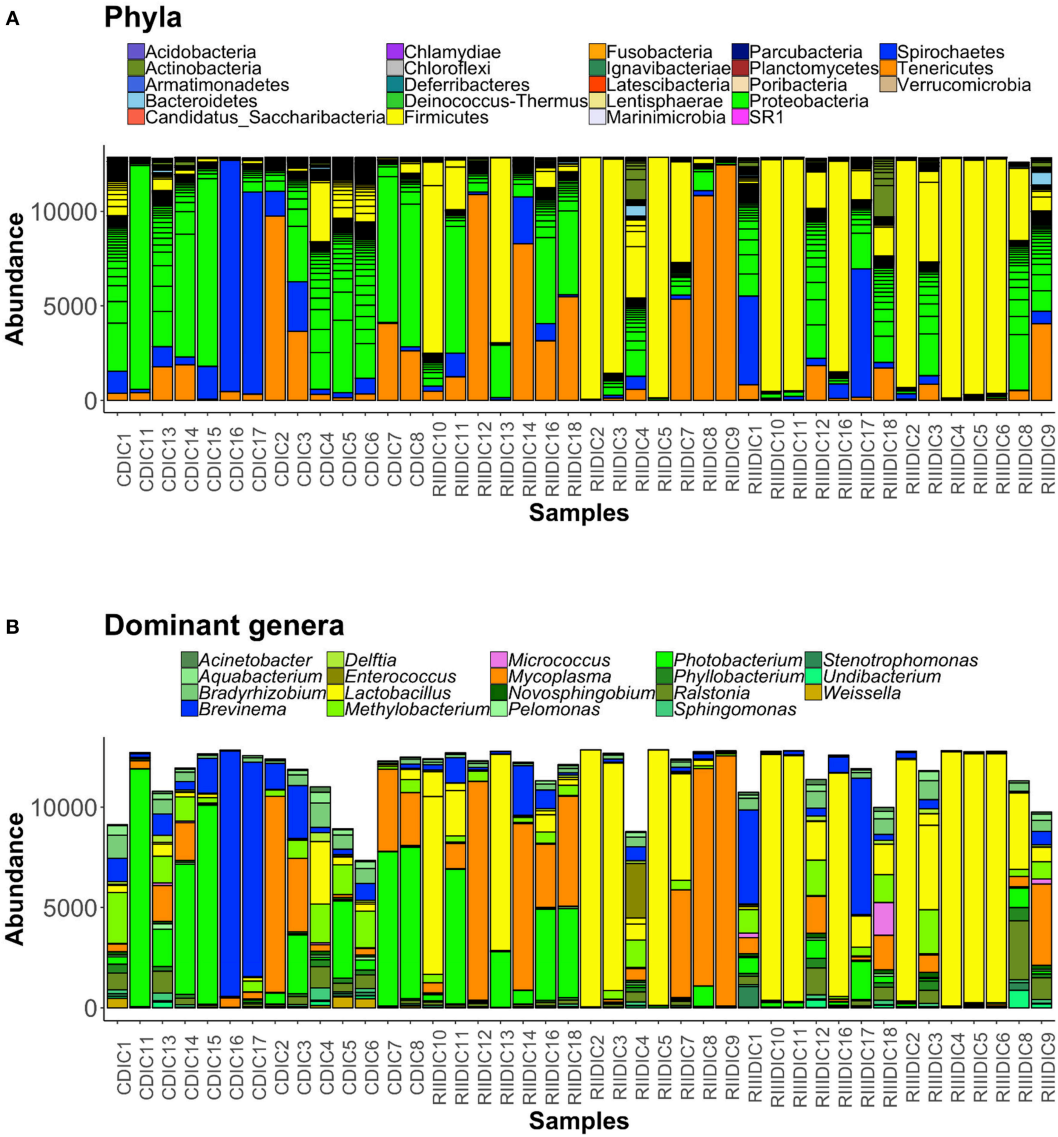
Barplots showing the abundance of all bacterial phyla **(A)**, and dominant genera **(B)**, in the intestinal content. The height of each bar segment represents the abundance of individual operational taxonomic units (OTUs) stacked in order from greatest to smallest, and separated by a thin black border line. Color codes for the dominant genera: Proteobacteria—shades of green, Spirochaetes—dark blue, Firmicutes—shades of yellow, Actinobacteria—orchid, and Tenericutes—dark orange.

**Figure 6:**
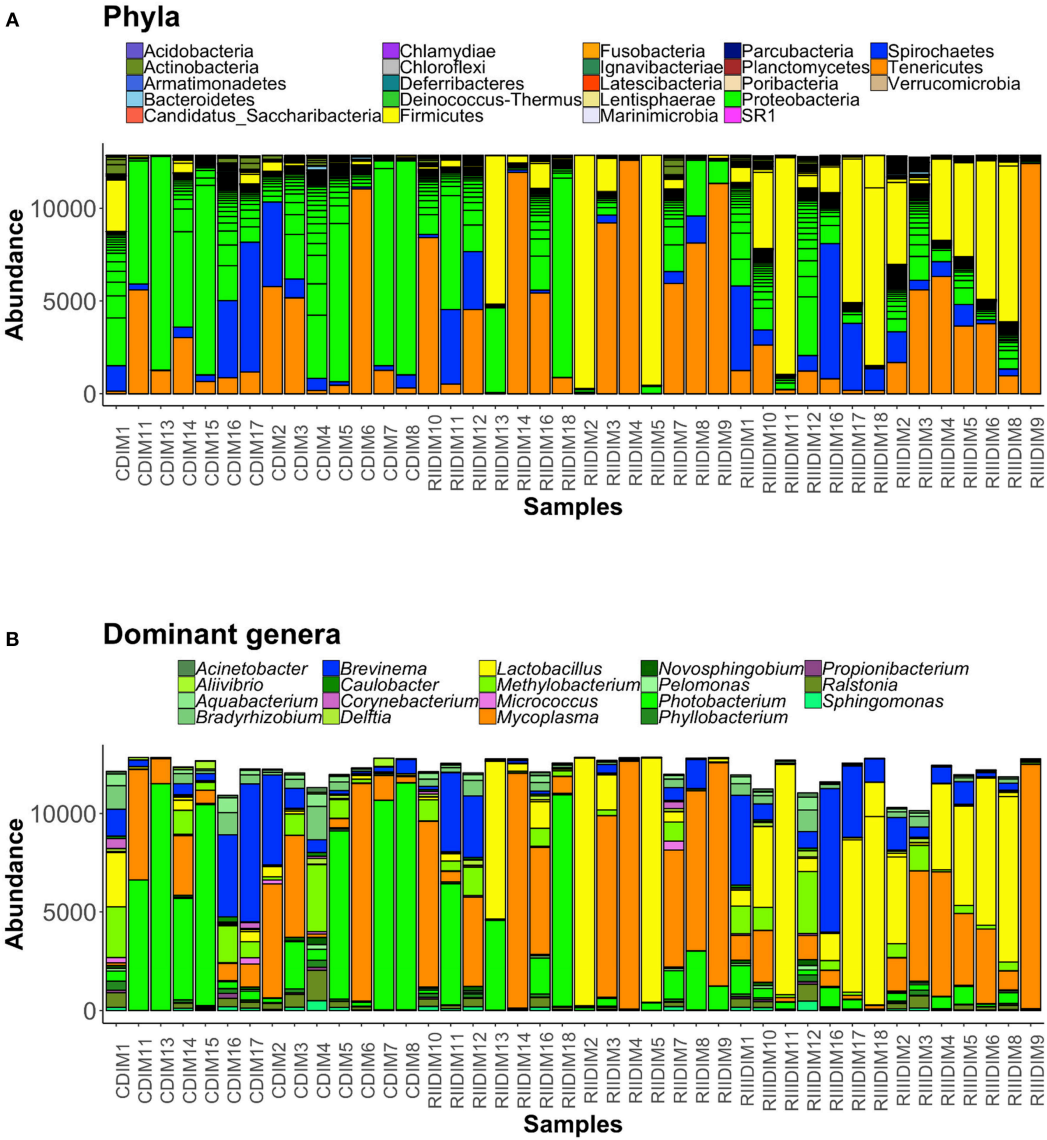
Barplots showing the abundance of all bacterial phyla **(A)**, and dominant genera **(B)** in the intestinal mucus. The height of each bar segment represents the abundance of individual operational taxonomic units (OTUs) stacked in order from highest to smallest, and separated by a thin black border line. Color codes for the dominant genera: Proteobacteria—shades of green, Spirochaetes—dark blue, Firmicutes—shades of yellow, Actinobacteria—shades of orchid, and Tenericutes—dark orange.

Bacteria belonging to 23 phyla were present in the DI content and mucus (Figures 5A, 6A). Firmicutes, Proteobacteria, Spirochaetes, Tenericutes, and Actinobacteria were found to be dominant in the intestine of the three study groups (Supplementary Figures 4A,C). Firmicutes were found to be more abundant than the rest, in both the content and mucus of the LAB-fed fish (Figures 5A,B and Figures 6A,B). The abundance of the phylum Tenericutes (content and mucus) was higher in RII-fed fish, than in the RIII-fed fish group (Figures 5A,B and Figures 6A,B). Proteobacteria (content and mucus) decreased in the LAB-fed groups compared to the control group (Figures 5A,B; Figures 6A,B and Table 1). The abundance of Spirochaetes was higher in the DI mucus of RIII-fed fish and lower in the RII-fed fish (Figures 6A,B). The abundant phyla in water is shown in Supplementary Figure 5A. The dominant phyla in water were Bacteriodetes and Proteobacteria (Supplementary Figure 5B). The changes in the abundance of most bacterial taxa in both DI content and mucus of the LAB-fed groups compared to the control group is shown in Table 1.

**Table 1:** Changes in abundances of the bacterial taxa by LAB feeding.

**Sample type**	**Intestinal content**		**Intestinal mucus**	
** 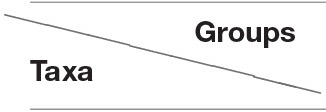 **	**RII**	**RIII**	**RII**	**RIII**
Acidobacteria	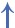	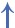	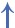	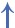
Actinobacteria	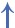	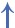	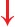	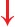
Fusobacteria	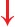	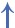	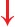	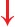
Deinococcus-Thermus	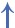	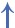	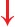	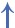
SR1	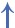	**–**	**–**	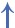
Chloroflexi	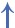	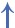	**–**	**–**
Parcubacteria	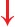	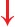	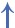	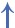
Planctomycetes	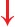	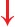	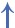	**–**
*Lactobacillus fermentum*	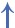	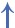	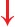	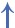
*Lactobacillus paraplantarum*	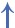	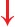	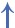	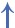
*Colwellia aestuarii*	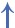	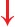	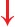	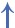
*Streptococcus sobrinus*	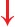	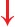	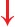	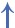
*Lewinella antarctica*	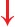	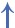	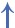	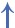
*Lactobacillus plantarum*	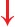	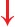	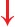	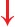
*Acinetobacter radioresistens*	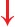	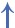	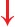	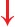
*Novosphingobium sediminicola*	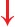	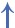	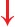	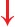
*Phyllobacterium myrsinacearum*	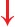	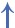	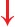	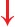
*Ralstonia pickettii*	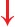	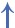	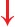	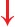
*Stenotrophomonas maltophilia*	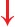	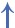	**TND**	**TND**
*Undibacterium oligocarboniphilm*	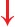	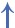	**TND**	**TND**
*Micrococcus luteus*	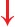	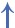	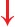	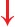
*Enterococcus cecorum*	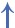	**–**	**TND**	**TND**
*Mycoplasma*	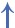	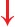	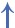	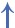
*Aquabacterium*	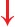	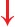	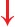	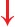
*Bradyrizhobium*	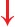	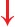	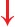	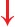
*Brevinema*	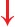	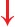	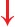	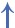
*Delftia*	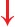	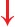	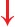	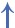
*Methylobacterium*	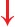	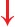	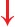	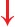
*Aquabacterium parvum*	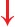	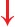	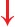	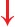
*Pelomonas*	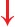	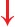	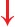	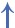
*Photobacterium*	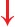	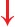	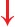	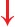
*Sphingomonas*	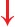	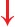	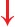	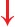
*Weissella*	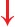	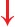	**TND**	**TND**
*Brevinema andersonii*	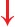	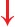	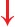	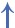
*Pelomonas saccharophila*	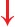	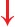	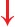	**–**
*Bradyrizhobium jicamae*	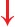	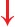	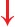	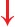
*Methylobacterium fujisawaense*	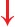	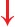	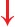	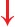
*Photobacterium phosphoreum*	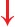	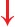	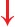	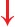
*Aliivibrio logei*	**TND**	**TND**	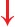	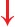
*Caulobacter segnis*	**TND**	**TND**	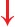	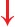
*Cornybacterium*	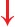	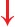	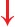	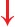
*Propionibacterium acnes*	**TND**	**TND**	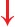	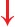

*Arrows indicate changes in abundance (blue arrow: increase, red arrow: decrease, bold black line: no change, TND: taxon not dominant)*.

At the genus level, *Lactobacilli* (*Lactobacillus fermentum* and *Lactobacillus paraplantarum*) were found to be the most dominant bacteria in the content and mucus of LAB-fed fish (Figure 5B, and Supplementary Figures 4B,D) and Mycoplasma was also found to be dominant in the DI mucus of LAB-fed fish (Figure 6B).

### Core Bacterial Communities of the Intestinal Microbiota

**Figure 7:**
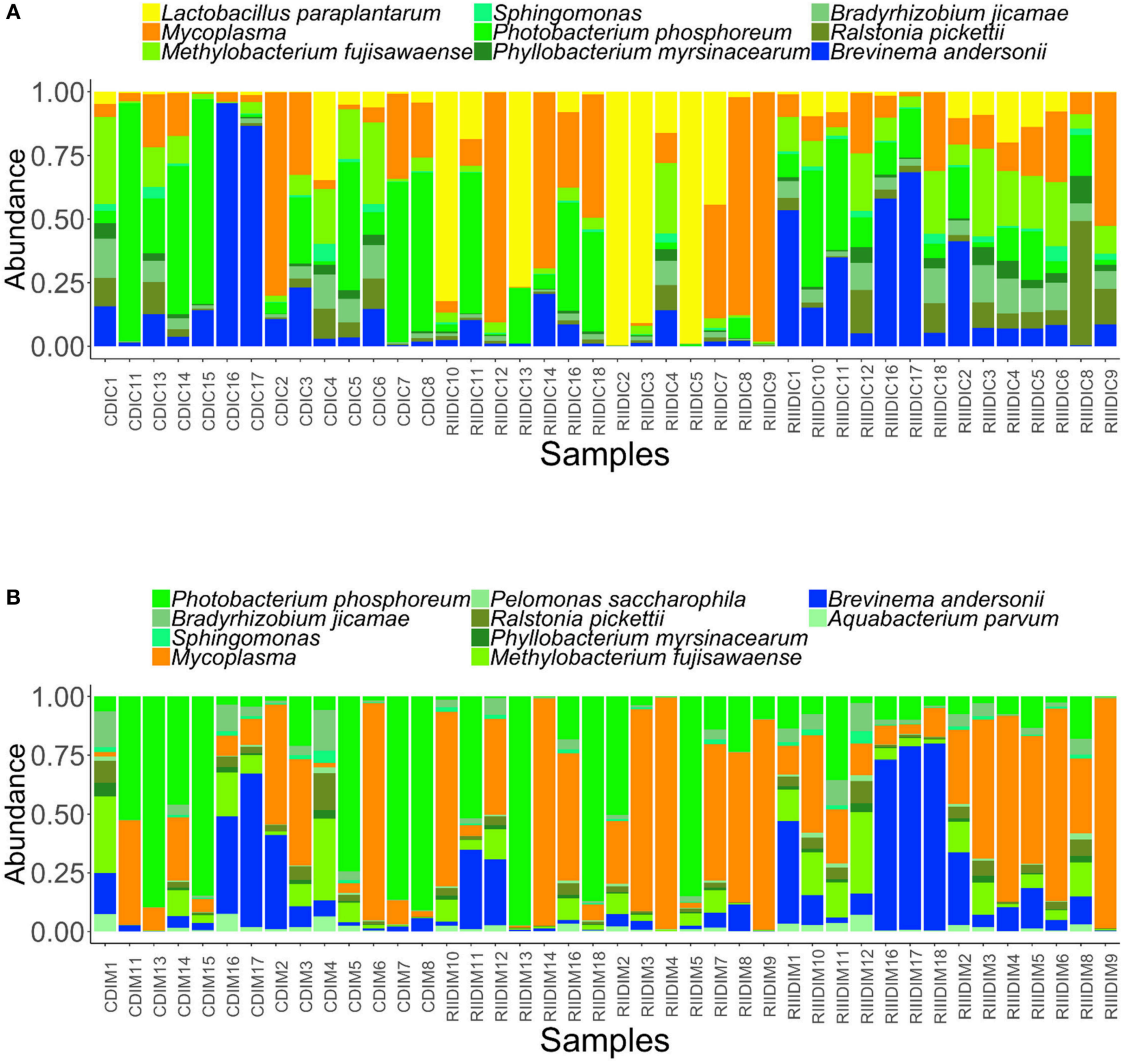
Abundance of the core bacterial taxa in the intestinal content **(A)** and mucus **(B)**. Color codes: Shades of green—Proteobacteria, yellow—Firmicutes, dark blue—Spirochaetes, and dark orange—Tenericutes.

We identified the core microbiota, i.e., the members of the bacterial communities that were commonly shared among 99% of the samples.The common core taxa–at prevalence (bacterial community population frequency) of 99% and abundance detection threshold of 20%–are shown in Figures 7A,B. In the DI content, the abundant genera in the LAB-fed fish, namely *Lactobacillus, Ralstonia (L. paraplantarum, R. pickettii)* and *Mycoplasma* were noted to be among the core bacterial members. *Bradyrizhobium, Photobacterium, Phyllobacterium, Brevinema, Methylobacterium* (*B. jicamae, P. phosphoreum, P. myrsinacearum, B. andersonii, M. fujisawaense*), and *Sphingomonas* were also the shared core taxa in the content (Figure 7A). In the DI mucus, the genera that had higher abundance in the RIII-fed fish viz. *Brevinema* and *Pelomonas* (*B. andersonii, P. saccharophila*) were observed among the core bacterial members. *Photobacterium, Ralstonia, Aquabacterium, Bradyrizhobium, Methylobacterium, Phyllobacterium*, (*P. phosphoreum, R. pickettii, A. parvum, B. jicamae, M. fujisawaense, P. myrsinacearum*), *Sphingomonas, and Mycoplasma* were also the shared core taxa of the intestinal mucus (Figure 7B).

The DPCoA indicated differences in the core members of the LAB-fed and the control group (content: F-statistic: 3.879, *R*^2^ = 0.165, *p* = 0.004; mucus: F-statistic: 5.844, *R*^2^ = 0.219, *p* = 0.001; Supplementary Figures 6A,B).

### Significantly Abundant and Relevant Bacterial Taxa of the Intestinal Microbiota

ANCOM analysis detected the significantly abundant bacterial OTU in the DI content, which turned out to be *L. fermentum* in RIII-fed fish (Table 1). However, this bacterium was not detected as a significant feature in the DI mucus.

Boruta analysis gave 9 and 8 relevant OTUs in the intestinal content and mucus, respectively. In the DI content, *L. fermentum, L. paraplantarum, Streptococcus sobrinus, Corynebacterium simulans, Lactococcus plantarum, W. cibaria, C. amphilecti*, and bacterial taxa belonging to *Streptococcus* and *Xanthomonodales* were the relevant bacteria. *L. paraplantarum* was found to be abundant in the RII-fed group, whereas *L. fermentum* and *Xanthomonodales* were found to be abundant in the RIII-fed group. *S. sobrinus, C. simulans, L. plantarum, W. cibaria, C. amphilecti* were reduced in abundance in the LAB-fed groups. In the mucus, *Lewinella antarctica, L. paraplantarum, L. fermentum, Salinisphaera, Colwellia aestuarii* and bacteria belonging to- *Gammaproteobacteria, Rhodobacteraceae*, and *Clostridiales* were found to be the relevant bacterial taxa (most of them were abundant in the mucus of the RIII-fed fish–Table 1).

### Association Network of OTUs

#### The DI Content Bacteria

**Figure 8:**
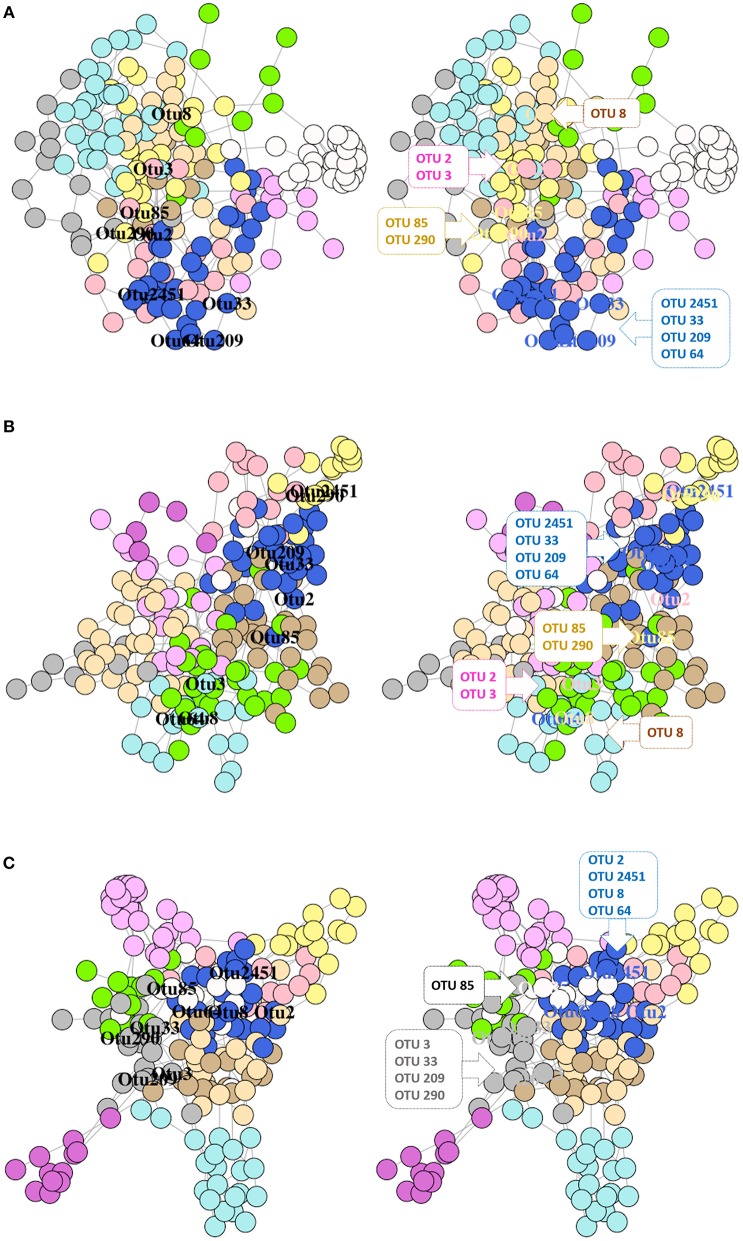
Network graphs showing the significantly abundant and relevant OTUs of the intestinal content in different modules of the network. Bacterial networks of the control **(A)**, RII **(B)**, and RIII **(C)** fish. Nodes represent OTUs and specific colors of the modules reveal the membership of the significantly abundant and relevant OTUs. The left graph shows the location of the OTUs and in the right graph, the significantly abundant and relevant OTUs that belong to the same module are shown in callouts.

The single-domain bacterial (SDB) network derived from the DI content of the 3 groups comprised of one giant connected component (Supplementary Figure 7). The significantly abundant and relevant OTUs were labeled based on their membership in different modules (Figures 8A–C). The connectivity pattern of the significantly abundant and relevant OTUs in the phylum-level co-occurrence network is shown in Supplementary Figures 9 A–C. The average node degrees were 4.27 (SD: 3.44), 3.71 (SD: 1.52), 4.06 (SD: 2.48) for the control, RII- and RIII-fed fish, respectively. Similarly, the values for betweenness were 370 (SD: 369), 396 (SD: 351), 388 (SD- 391). The average node degrees and betweenness of the three groups were not significantly different. The degree of assortativity (assortativity coefficient *c*_*a*_*)* of the phylum-level network associated with the three groups (control, RII- and RIII-fed fish) were 0.09, 0.19, and 0.10, respectively. The significantly abundant and relevant OTUs belonged to different phyla and modules (Figures 8A–C and Supplementary Figures 9 A–C). The degree distribution of the microbial network (for all OTUs) of the study groups (Supplementary Figure 11A) revealed that there are many highly connected hub nodes for the bacterial network of the RII-fed fish and the hubs of the control group have more node degrees.

#### The DI Mucus Bacteria

**Figure 9:**
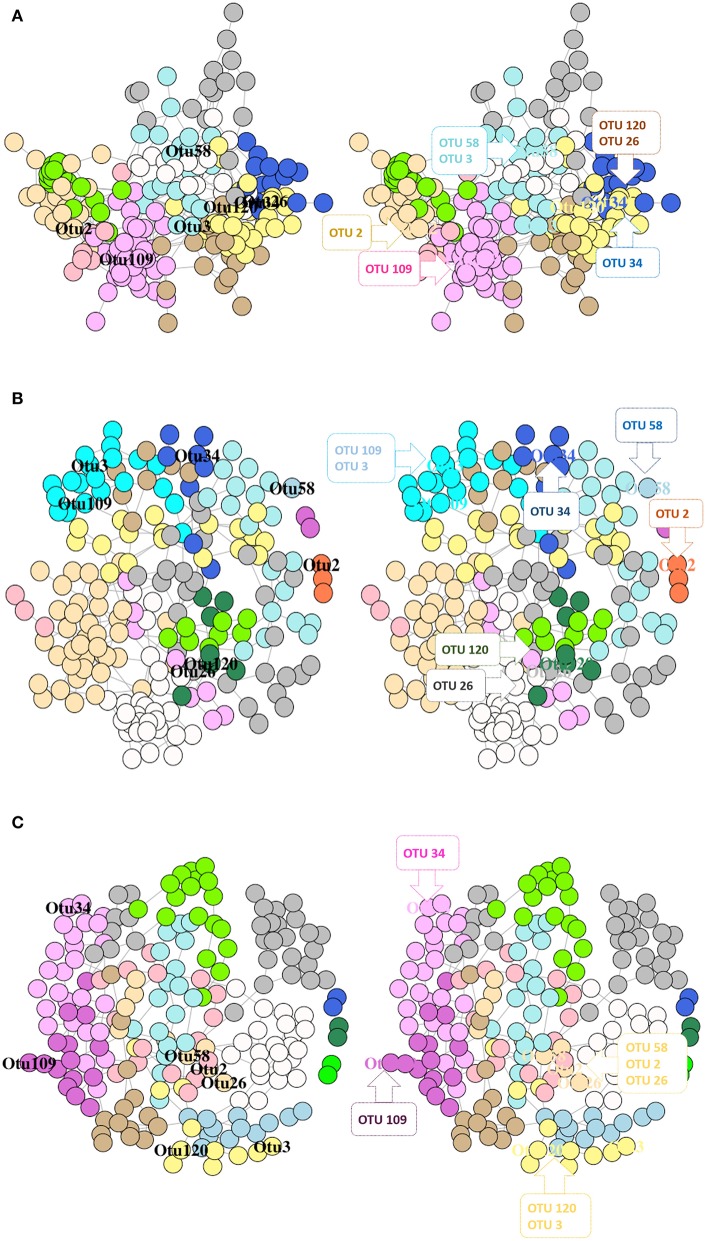
Network graphs showing the significantly abundant and relevant OTUs of DI mucus in different modules based on their membership for control fish **(A)**, RII **(B)**, and RIII fish **(C)**. Nodes represent OTUs and specific colors of the modules reveal the membership of the significantly abundant and relevant OTUs. The left graph shows the location of the OTUs and in the right graph the significantly abundant and relevant OTUs that belong to the same module are shown in callouts.

The SDB network derived from the DI mucus of the control, RII, and RIII groups comprised of one giant connected component (Supplementary Figure 8). In the bacterial network of RII-fed fish, we observed a singleton (*C. aestuarii*), a dyad (2 OTUs of *Mycoplasma*), and a triad (*L. paraplantarum, W. cibaria*, and *P. piscicola*) with no connection to the main network (Supplementary Figure 8). As for the RIII-fed group, there were 3 dyads (Sphingobacteriales + Myxococcales, 2 OTUs of *Mycoplasma*, and Xanthomonadales + Gammaproteobacteria) with no connection to the main network (Supplementary Figure 8). The significantly abundant and relevant OTUs were labeled based on their membership in different modules (Figures 9A–C).

The connectivity pattern of the significantly abundant and relevant OTUs in the phylum-level co-occurrence network is shown in Supplementary Figures 10 A–C. The average node degrees were 4.12 (SD: 2.20), 2.29 (SD: 2.09), 2.74 (SD: 1.19) for the control, RII- and RIII-fed fish, respectively. The values for betweenness of the control, RII- and RIII-fed fish were 505 (SD: 664), 481 (SD: 596), 613 (SD: 766), respectively. Dunn's test identified significant differences between the LAB-fed groups, and between control and RIII-fed fish; for node degree, but not for edge betweenness; *p* = 0.0002, *p* = 0.003 and *p* = 0.08, *p* = 0.07, respectively. The degree of assortativity (assortativity coefficient *c*_*a*_*)* of the phylum-level network for the three groups (control, RII- and RIII-fed fish) were −0.01, −0.07, and 0.13, respectively. The degree distribution of the microbial network (for all OTUs) of the three groups is shown in Supplementary Figure 11B. The node degree histogram showed that the hubs of the RII-fed groups have higher node degrees than the other groups.

**Figure 10:**
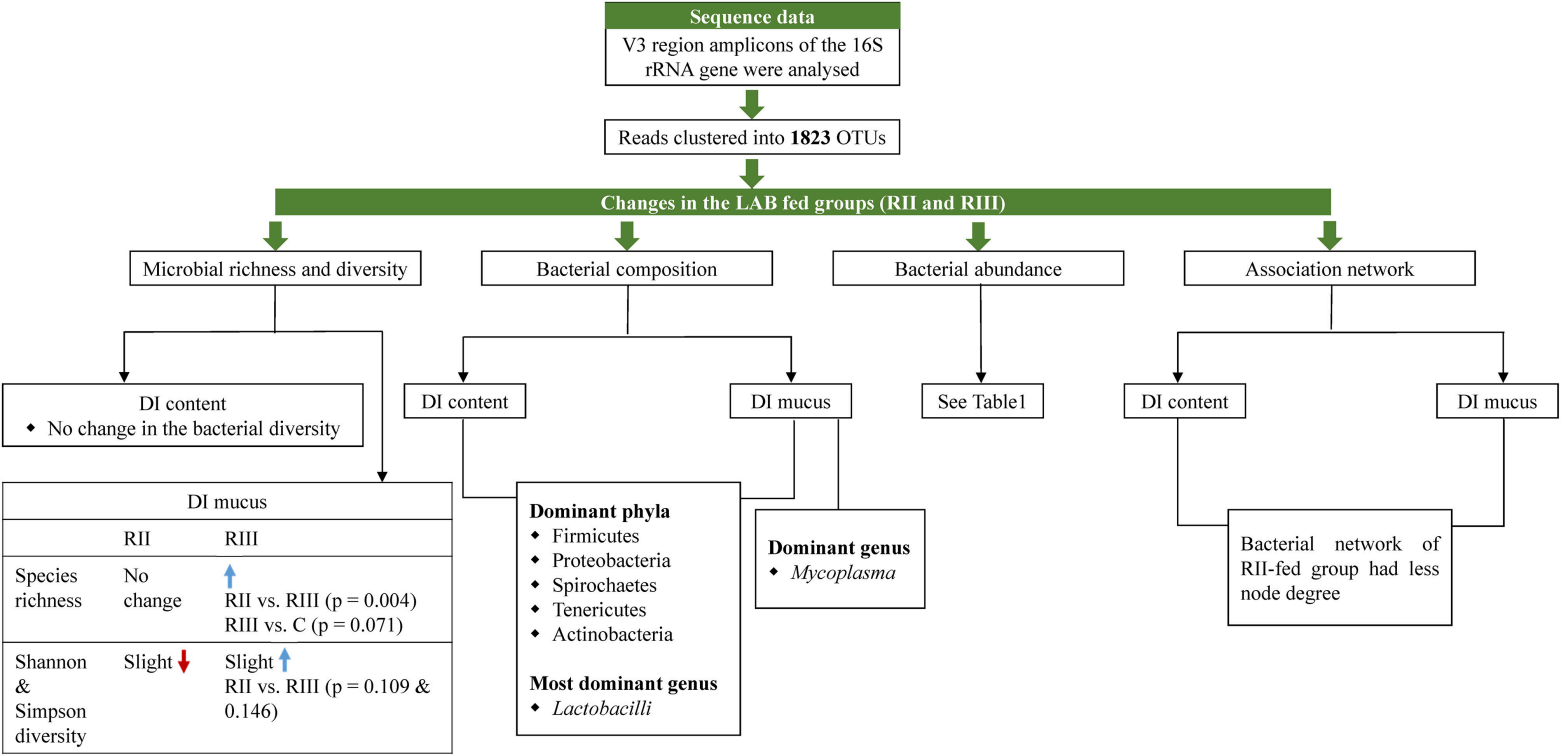
Illustration summarizing the salient observations of the study. DI, distal intestine; C, control group; RII and RIII, LAB-fed groups.

The main results of this study are summarized in Figure 10.

## Discussion

Probiotics are live microbes that can impart health-benefiting effects on host organisms. For instance, feeding of some species belonging to genera *Lactobacillus* and *Bifidobacterium* can elicit positive effects on host health (Wang et al., 2015; Bagarolli et al., 2017). Probiotics alter the gut microbiota and interact with them to produce several types of metabolites, vitamins, and antimicrobial agents that affect the host physiology (Saulnier et al., 2011; O'Shea et al., 2012; LeBlanc et al., 2017). In the present study, we investigated the intestinal microbiota changes in Atlantic salmon after feeding them with dietary supplements of two *Lactobacillus* spp., named RII and RIII. To understand the differences in the microbial community associated with the content and mucus of the DI, the bacteria in the two samples were analyzed separately because the microbial niche in the DI mucus is distinct compared to the intestinal contents.

Feeding LAB to the fish may facilitate their establishment in the intestine, although significant difference was noted for the abundance of only one of the two LAB species. The feed-delivered organisms also altered the diversity and composition of the DI bacteria differently. RIII supplementation caused a significant increase in the species richness and phylogenetic diversity of the bacterial community in DI mucus. Furthermore, both RII and RIII caused a shift in the community composition; bacteria belonging to different genera were altered in the two feed groups. The co-occurrence networks indicated differential bacterial associations in the control and LAB-fed groups.

Water bacterial communities may have an effect on the microbiota of fish. To clarify this, we compared the microbial community composition in the intestinal and environmental samples. Notwithstanding the fact that different extraction methods can cause small variations in the microbial profile (Wagner Mackenzie et al., 2015) studies have shown that rearing water has a minor effect on the GI microbiota in fish (Giatsis et al., 2015; Uren Webster et al., 2018). Betiku et al. (2018) have demonstrated that recirculating water systems have more diverse microbial composition compared to the flow-through system. However, similar to other reports (Yan et al., 2016, Lokesh et al., 2018, Gupta et al., under review) water bacterial communities might not have affected the intestinal bacterial profile in our study. Also, none of the dominant OTUs of water were detected in the DI of fish, suggesting that host-specific gut microbial species selection is modulated by the host gut habitat and host's genotype (Giatsis et al., 2015).

### LAB Increases the Microbial Diversity in the Intestinal Mucus

Corresponding to our observation on the content bacteria, a few previous studies have also shown that LAB supplementation does not alter the intestinal bacterial diversity (Chao1 and Shannon diversities); in humans (Van Zanten et al., 2014) and in mice with colon cancer (Mendes et al., 2018). On the other hand, species richness, Shannon and Simpson diversities, and PD of the bacteria in the DI mucus were higher in the RIII-fed fish. In the case of mucus bacteria of RII-fed fish, we noted a slight decrease (*p* > 0.05) in alpha diversity compared to the control fish. Previous studies have shown that *Lactobacillus* can increase the bacterial PD in the gut of mice (Usui et al., 2018) and weaning piglets (Zhao et al., 2016). On the contrary, offering LAB in combination with *Bifidobacterium breve* and *Bifidobacterium longum* did not result in greater bacterial species diversity (Chao1, Shannon index and PD) in mice that received antibiotics (Grazul et al., 2016).

### LAB Promotes the Abundance and Dominance of Intestinal *Lactobacillus* and Other Firmicutes

*L. paraplantarum* (LP) is related to *L. plantarum* (Curk et al., 1996). It was dominant in the RII-fed group and *L. fermentum* (LF) was found dominant in the RIII-fed group. *Lactobacilli* are a group of gram-positive ubiquitous LAB that produce organic acids as end products of their metabolic activity linked to carbohydrate fermentation (Bernardeau et al., 2006). LP is known to produce bacteriocins, which are antimicrobial peptides produced as a defense response (Tulini et al., 2013). A *Lactobacillus* isolate (LP 11-1) stimulated the innate immune system and induced tolerance against the pathogenicity of *Pseudomonas aeruginosa* in silkworm (Nishida et al., 2017). LF has been found to restore the expression of markers associated with the maintenance of intestinal barrier function, and recover the SCFAs- and lactic acid-producing bacterial populations in mouse suffering from colitis (Rodriguez-Nogales et al., 2017).

*Lactobacillus* is part of the normal intestinal flora of fish (Ringø et al., 1995; Spanggaard et al., 2000; Ringø and Olsen, 2003). In zebrafish, probiotic *Lactobacillus* helps to overcome infection (He et al., 2017). In Nile tilapia (*Oreochromis niloticus*), LF is known to improve fish immune response (Nwanna and Bamidele, 2014). LF (LbFF4 strain) along with *L. plantarum* (LbOG1 strain) exhibit *in vitro* antibacterial activities against fish pathogens in *Clarias gariepinus* (Adenike and Olalekan, 2009). The higher abundance of intestinal *Lactobacillus* members and the altered bacterial abundance in the LAB-fed fish confirms that LAB feeding can change the intestinal microbial composition.

*Enterococcus cecorum*, was also found to be dominant in the content of the RII-fed group compared to the control group (Table 1). *Enterococcus* spp. isolated from the intestine of rainbow trout (*Oncorhynchus mykiss*) are used as probiotics due to their antimicrobial activity against fish pathogens (Carlos et al., 2015). The functional potential of *E. cecorum* in Atlantic salmon has not yet come to light although one particular strain is known to cause infections in broilers (Herdt et al., 2009).

Clostridiales (belonging to Firmicutes) were higher in the mucus of salmon offered diets with RIII. Commensal Clostridiales are known to promote gut health by modulating gut homeostasis and taking part in immune activation (Lopetuso et al., 2013).

### LAB Favors Certain Members of Tenericutes, Spirochaetes, and Actinobacteria

LAB significantly aided in altering the abundance of the genus *Mycoplasma* (Tenericutes) and *B. andersonii* (Spirochaetes) in the mucus, which are the common core members in the DI content of Atlantic salmon (Figure 7A). *Mycoplasma* has consistently been isolated from salmon intestine (Holben et al., 2002; Zarkasi et al., 2014) and its presence as a core microbiota suggests that it may be a commensal organism in the intestinal ecosystem. *B. andersonii* has been reported in the intestinal microbiota of flatfish, *Solea senegalensis* (Tapia-Paniagua et al., 2010). Although *B. andersonii* is known to digest lignocellulose and fix nitrogen in termite guts (Kudo, 2009), their functional importance needs to be elucidated. The abundance of the genus *Micrococcus* (*M. luteus*), a member of Actinobacteria, was higher in the DI content of the RIII-fed group (Table 1). Though *M. luteus* is known to be a pathogen for rainbow trout (*Salmo trutta L*.) and brown trout (*Oncorhynchus mykiss*) (Pkala et al., 2018) an *in vivo* feeding study has suggested that they can enhance the growth and health of Nile tilapia (Abd El-Rhman et al., 2009).

### LAB Largely Decreased the Abundance of Proteobacteria

Proteobacteria is the most abundant phylum in many marine and freshwater fishes (Yan et al., 2016; Lokesh et al., 2018) and it is also known to dominate the gut microbiota of Atlantic salmon (Gajardo et al., 2016; Lokesh et al., 2018). Therefore, it was surprising to find this taxon in low abundance in the LAB-fed and the control fish. A general decrease in the abundance of intestinal Proteobacteria has also been reported in farmed Atlantic salmon that were transferred to seawater (Rudi et al., 2018). Taxa belonging to Proteobacteria are involved in metabolic pathways that participate in carbon and nitrogen fixation and in the stress response regulatory system (Vikram et al., 2016). They are also important in the digestive process in fish (Romero et al., 2014). *P. phosphoreum*, a known gut symbiont of marine fish, helps in chitin digestion and use luciferase- reoxidize reduced coenzymes and other molecules for metabolism (Nealson and Hastings, 1979). *N. sediminicola* and *P. myrsinacearum* are known as nitrogen-fixing bacteria (Gonzalez-Bashan et al., 2000; Muangthong et al., 2015). On the other hand, *R. pickettii* formerly known as *Burkholderia pickettii* has genes to biodegrade aromatic hydrocarbons (Ryan et al., 2007). In the current and in our recent (Gupta et al, under review) studies we found that *P. myrsinacearum* and *R. pickettii* are part of the core gut microbiota of Atlantic salmon; *N. sediminicola* was also significantly abundant in the intestinal mucus of the fish fed oligosaccharide. Functions of the aforementioned bacteria are not yet reported in fish.

### LAB Affects the Microbial Association

We inferred single-domain networks using the SPEIC-EASI framework, and highlighted the significantly abundant and relevant OTUs in the intestinal microbiota. For DI mucus, the inferred SDB network for RII-fed fish showed lower overall connectivity. The node degree histograms also communicate interesting information about the network; the mucus bacteria of RII-fed group had hubs with more node degree. However, the lower average node degree and lower selective linking of the RII-fed group indicate less interactions among the gut bacteria. Cooperative microbial communities are known to provide microbiome stability because of their functional dependence. Studies have shown that the stability declines with an increase in microbial diversity and proportion of cooperative interactions (Coyte et al., 2015). However, higher cooperating microbial communities can cause a runaway effect that can collapse the competing microbial population due to over-representation of the most stable community (McNally and Brown, 2016).

The dyads in the mucus bacterial networks of LAB-fed fish were different, the exception being the one constructed with 2 OTUs of *Mycoplasma* which had higher abundance in the RII-fed fish and lower abundance in the RIII-fed fish. This result could be suggesting that intestinal *Mycoplasma* in the LAB-fed fish was not associated with other gut bacterial communities. In the content of LAB-fed fish, most of the labeled OTUs (except OTU 8) were existing in their respective modules (Figures 8B, C). In the mucus of RIII-fed fish OTUs belonging to *C. aestuarii, L. paraplantarum* and Clostridiales were found to exist in one module. Clostridiales and Rhodobacteraceae, which had same module membership in the network of the control fish were no longer closely associated after LAB feeding. So was the case with *L. fermentum* and *C. aestuarii*. Members affiliated to Rhodobacteraceae are known for their denitrification properties, and Kraft et al. (2014) have shown that Clostridiales indirectly participates in nitrate respiration by providing fermentation substrates (e.g., acetate, formate, or hydrogen) to Rhodobacteraceae-like denitrifiers. Our findings suggests that the taxa belonging to the same module can be functionally dependent but the alteration of their membership after LAB feeding has to be further investigated.

The mucus bacteria of RIII-fed fish had higher species richness and PD, and the significantly abundant and relevant OTUs belonged to different modules. For the RIII-associated network, 2 OTUs each belonging to two modules (Rhodobacteraceae and *L. fermentum*; *C. aestuarii*, and Clostridiales) had higher abundances compared to the control group. In addition, significantly abundant and relevant bacteria had higher abundance in the RIII-fed fish compared to the control group. This abundance pattern does not indicate negative feedback loops (Coyte et al., 2015). These results of bacterial networks have to be validated through culture-based studies.

## Conclusion

In summary, LAB feeding promoted the dominance of intestinal *Lactobacillus* (Firmicutes) and certain members of the phyla Tenericutes, Spirochaetes, and Actinobacteria. Although the abundances of many members of Proteobacteria were decreased, the phylum remained dominant in the distal intestine of Atlantic salmon. Dietary supplementation with the two LAB strains shifted the intestinal bacterial community composition. Furthermore, the co-occurrence networks of the intestinal bacteria were also different for the LAB-fed fish. Taken together, our results show that the LAB influences the gut microbiota of Atlantic salmon. This information will help in future studies that explore the microbial interactions between LAB-modulated gut microbiota and the host.

## Author Contributions

MS and VK procured the funding for the study. VK, MS, JK, AF, and SG designed the study. JK provided the probiotics. AF and SG conducted the feeding experiment. SG performed the 16S rRNA sequencing studies. SG, VK, and JF analyzed the data. SG wrote the manuscript with the guidance of VK. All authors read, revised and approved the manuscript.

### Conflict of Interest Statement

The authors declare that the research was conducted in the absence of any commercial or financial relationships that could be construed as a potential conflict of interest.

## Supplementary Material

Supplementary Figure 1 Sample-size-based rarefaction curves for the reads obtained from the intestinal content **(A)** and mucus **(B)**. The shaded portion around each line represents the 95% confidence interval. Color code for the feed groups: green lines- control, orange lines- RII, pink lines- RIII. Codes for content samples: CDM-control, RIIDC-RII, RIIIDC-RIII. Codes for mucus samples: CDM-control, RIIDM-RII, RIIIDM-RIII.

Supplementary Figure 2 Double principal coordinate analysis plots showing the beta diversity of the bacterial communities. Tank and inlet water **(A)**, control intestinal content and control tank water: F-statistic = 4.035, *R*^2^ = 0.211, *P* = 0.01 **(B)**, RII intestinal content and RII tank water: F-statistic = 2.375, *R*^2^ = 0.136, *P* = 0.07 **(C)**, RIII intestinal content and RIII tank water: F-statistic = 5.006, *R*^2^ = 0.250, *P* = 0.002 **(D)**, Control intestinal mucus and control tank water: F-statistic = 16.291, *R*^2^ = 0.520, *P* = 0.003 **(E)**, RII intestinal mucus and RII tank water: F-statistic = 2.934, *R*^2^ = 0.163, *P* = 0.051 **(F)**, RIII intestinal mucus and RIII tank water: F-statistic = 3.910, *R*^2^ = 0.206, *P* = 0.03 **(G)**.

Supplementary Figure 3 Double principal coordinate analysis plots showing the beta diversity of the bacterial communities. Tank biofilm bacteria **(A)**, Control intestinal content and control tank biofilm: F-statistic = 2.061, *R*^2^ = 0.120, *P* = 0.082 **(B)**, RII intestinal content and RII tank biofilm: F-statistic = 1.915, *R*^2^ = 0.113, *P* = 0.015 **(C)**, RIII intestinal content and RIII tank biofilm: F-statistic = 4.171, *R*^2^ = 0.217, *P* = 0.043 **(D)**, Control intestinal mucus and control tank biofilm: F-statistic = 5.807, *R*^2^ = 0.1279, *P* = 0.002 **(E)**, RII intestinal mucus and RII tank biofilm: F-statistic = 1.476, *R*^2^ = 0.09, *P* = 0.146 **(F)**, RIII intestinal mucus and RIII tank biofilm: F-statistic = 2.078, *R*^2^ = 0.121, *P* = 0.076 **(G)**.

Supplementary Figure 4 Barplots showing the dominant bacterial phyla and species in the intestinal content **(A,B)** and mucus **(C,D)**.

Supplementary Figure 5 Barplots showing the abundance of the bacterial phyla **(A)**, dominant phyla **(B)** in the tank water. The height of each bar segment represents the abundance of individual operational taxonomic units (OTUs) stacked in order from largest to smallest, and separated by a thin black border line. Color codes: Proteobacteria—green, Bacteroidetes—light blue.

Supplementary Figure 6 DPCoA showing the differences in the composition of the core members of the intestinal content **(A)** and mucus **(B)** samples of the control and LAB-fed groups.

Supplementary Figure 7 The single-domain network graph of the bacteria in the intestinal content. Nodes represent different phyla shown in different colors. The three panels represent the three feed groups: Control **(A)**, RII **(B)**, RIII **(C)**.

Supplementary Figure 8 The single-domain network graph of the bacteria in the intestinal mucus. Nodes represent different phyla shown in different colors. The three panels represent the three feed groups: Control **(A)**, RII **(B)**, RIII **(C)**.

Supplementary Figure 9 Network association graph showing the connectivity pattern of the significantly abundant and relevant OTUs in the intestinal content of the Control **(A)**, RII **(B)**, and RIII **(C)** groups.

Supplementary Figure 10 Network association graph showing the connectivity pattern of the significantly abundant and relevant OTUs in the intestinal mucus of the Control **(A)**, RII **(B)**, and RIII **(C)** groups.

Supplementary Figure 11 Histograms showing the degree distribution of the bacterial networks associated with the intestinal content **(A)** and mucus **(B)**.
