# Corrigendum: Lactobacillus Dominate in the Intestine of Atlantic Salmon Fed Dietary Probiotics

**DOI:** 10.3389/fmicb.2019.01094

**Published:** 2019-05-15

**Authors:** Shruti Gupta, Adriána Fečkaninová, Jep Lokesh, Jana Koščová, Mette Sørensen, Jorge Fernandes, Viswanath Kiron

**Affiliations:** ^1^Faculty of Biosciences and Aquaculture, Nord University, Bodø, Norway; ^2^Department of Food Hygiene and Technology, University of Veterinary Medicine and Pharmacy in Košice, Košice, Slovakia; ^3^Department of Microbiology and Immunology, The University of Veterinary Medicine and Pharmacy in Košice, Košice, Slovakia

**Keywords:** fish, *Salmo salar*, feed additive, probiotics, intestinal bacteria, *Lactobacillus*, microbiota, amplicon sequencing

In the original article, there were mistakes in Table 1 as published. NA was stated as “not present” in the footnote of Table 1 of the original article. For some of the taxa in the table this was not true. Therefore, we replaced NAs with up or down arrows, and indicated TND as taxon not dominant. The corrected Table 1 appears below. The authors apologize for this error and state that this does not change the scientific conclusions of the article in any way. The original article has been updated.

**Table 1:** Changes in abundances of the bacterial taxa by LAB feeding.

**Sample type**	**Intestinal content**		**Intestinal mucus**	
** 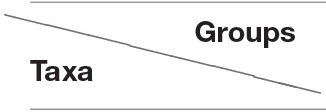 **	**RII**	**RIII**	**RII**	**RIII**
Acidobacteria	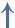	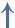	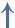	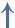
Actinobacteria	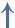	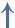	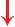	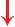
Fusobacteria	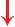	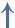	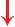	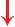
Deinococcus-Thermus	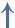	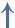	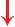	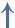
SR1	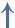	**–**	**–**	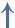
Chloroflexi	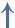	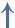	**–**	**–**
Parcubacteria	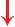	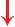	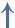	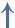
Planctomycetes	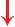	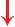	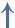	**–**
*Lactobacillus fermentum*	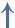	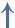	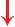	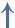
*Lactobacillus paraplantarum*	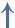	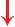	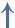	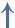
*Colwellia aestuarii*	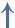	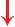	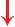	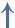
*Streptococcus sobrinus*	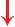	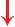	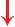	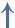
*Lewinella antarctica*	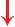	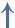	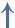	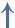
*Lactobacillus plantarum*	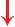	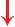	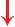	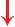
*Acinetobacter radioresistens*	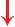	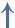	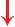	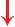
*Novosphingobium sediminicola*	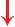	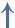	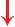	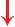
*Phyllobacterium myrsinacearum*	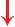	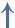	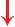	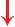
*Ralstonia pickettii*	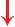	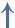	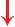	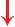
*Stenotrophomonas maltophilia*	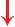	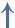	**TND**	**TND**
*Undibacterium oligocarboniphilm*	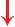	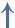	**TND**	**TND**
*Micrococcus luteus*	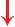	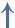	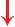	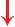
*Enterococcus cecorum*	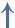	**–**	**TND**	**TND**
*Mycoplasma*	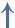	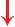	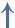	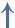
*Aquabacterium*	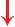	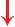	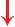	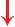
*Bradyrizhobium*	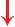	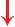	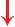	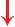
*Brevinema*	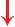	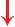	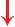	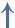
*Delftia*	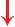	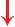	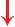	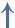
*Methylobacterium*	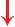	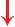	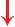	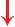
*Aquabacterium parvum*	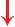	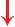	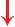	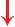
*Pelomonas*	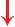	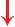	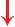	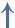
*Photobacterium*	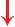	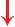	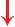	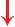
*Sphingomonas*	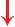	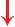	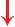	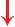
*Weissella*	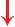	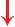	**TND**	**TND**
*Brevinema andersonii*	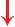	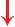	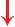	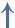
*Pelomonas saccharophila*	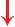	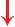	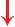	**–**
*Bradyrizhobium jicamae*	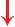	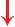	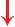	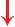
*Methylobacterium fujisawaense*	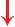	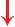	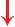	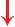
*Photobacterium phosphoreum*	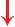	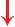	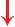	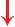
*Aliivibrio logei*	**TND**	**TND**	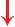	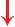
*Caulobacter segnis*	**TND**	**TND**	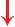	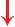
*Cornybacterium*	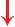	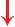	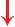	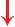
*Propionibacterium acnes*	**TND**	**TND**	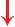	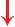

*Arrows indicate changes in abundance (blue arrow: increase, red arrow: decrease, bold black line: no change, TND: taxon not dominant)*.

