# Supplementary material for: Lactobacillus Dominate in the Intestine of Atlantic Salmon Fed Dietary Probiotics
Source: Front Microbiol. 2019 Jan 11;9:3247. doi: 10.3389/fmicb.2018.03247 (PMC6343587; doi:10.3389/fmicb.2018.03247)

## Supplementary Figure 1A

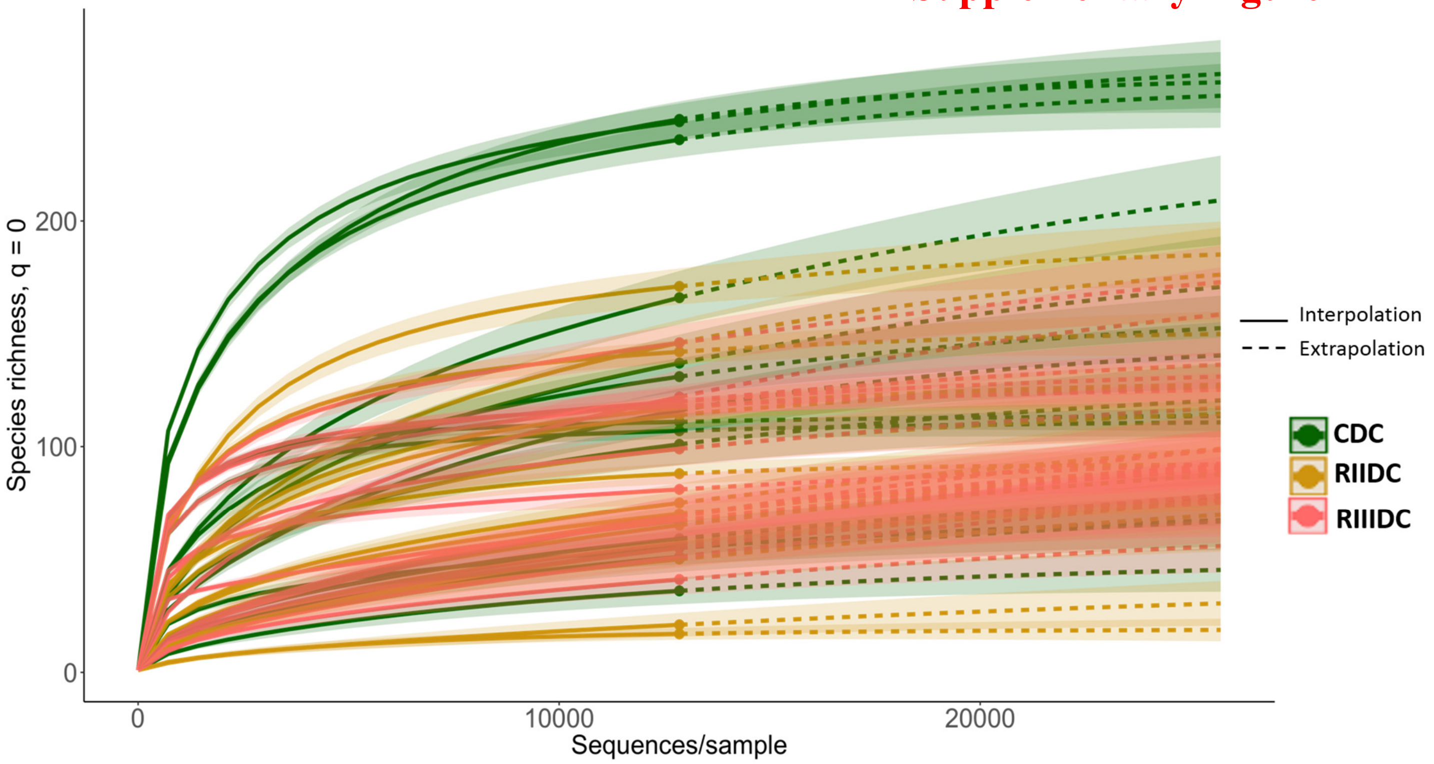


## Supplementary Figure 2A

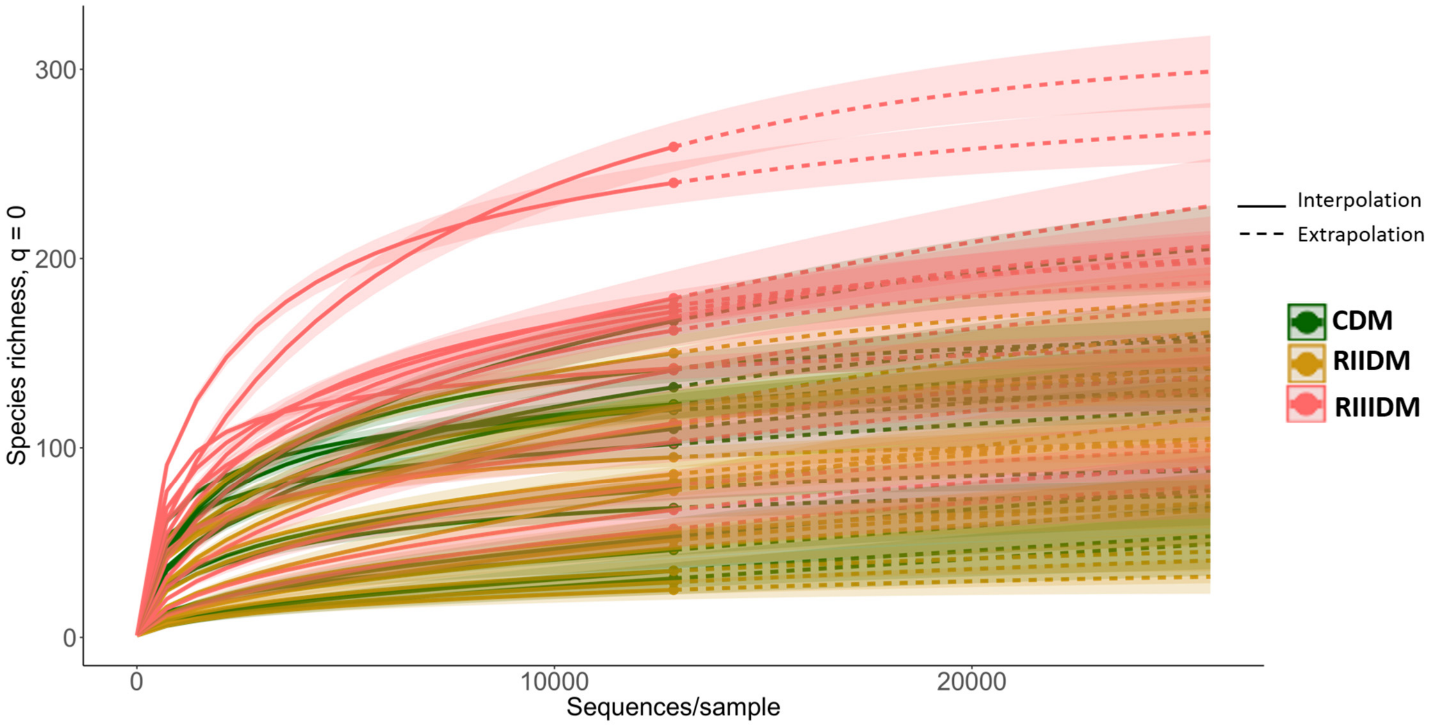

Supplementary Figure 2A

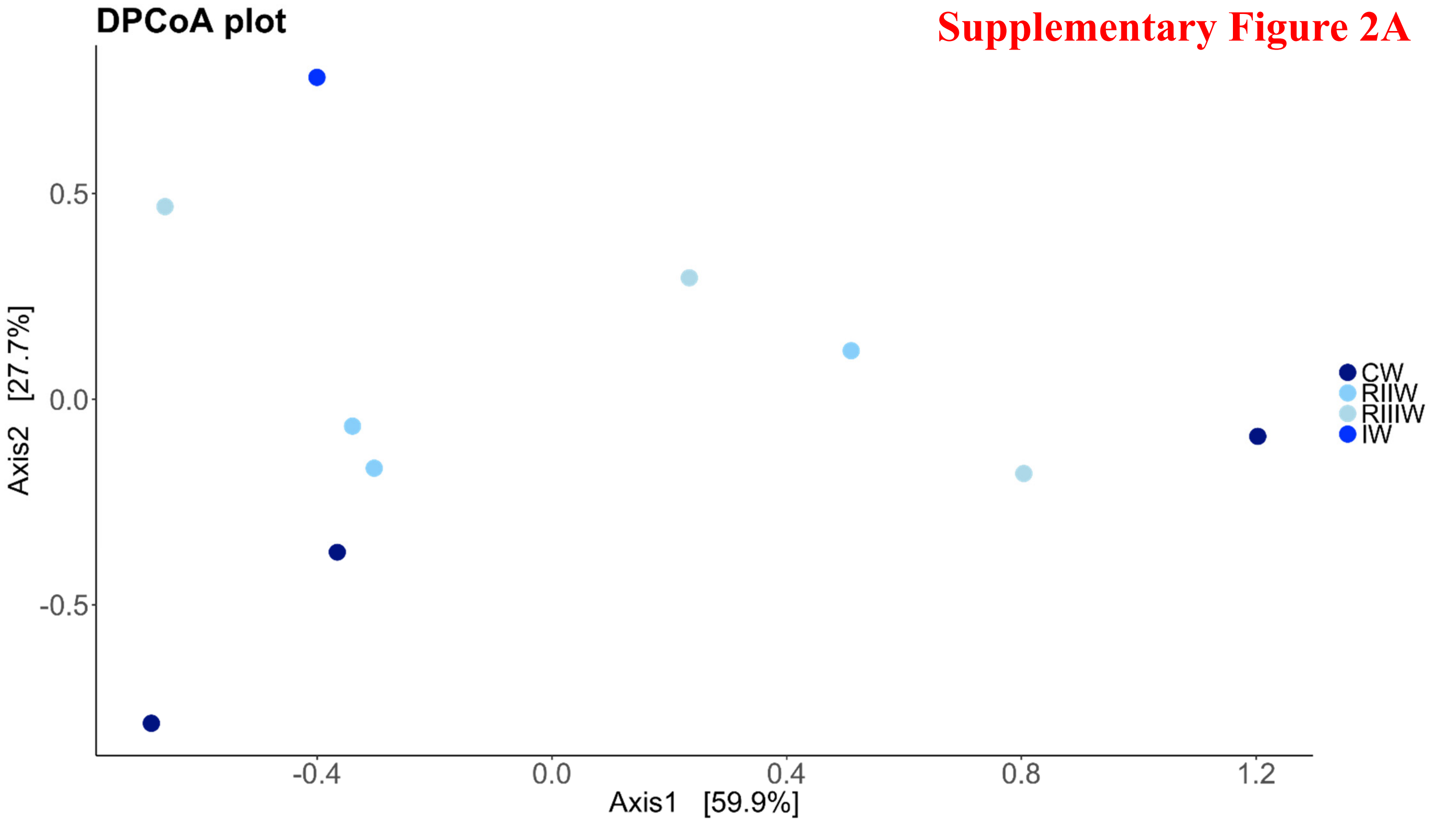


Supplementary Figure 2B

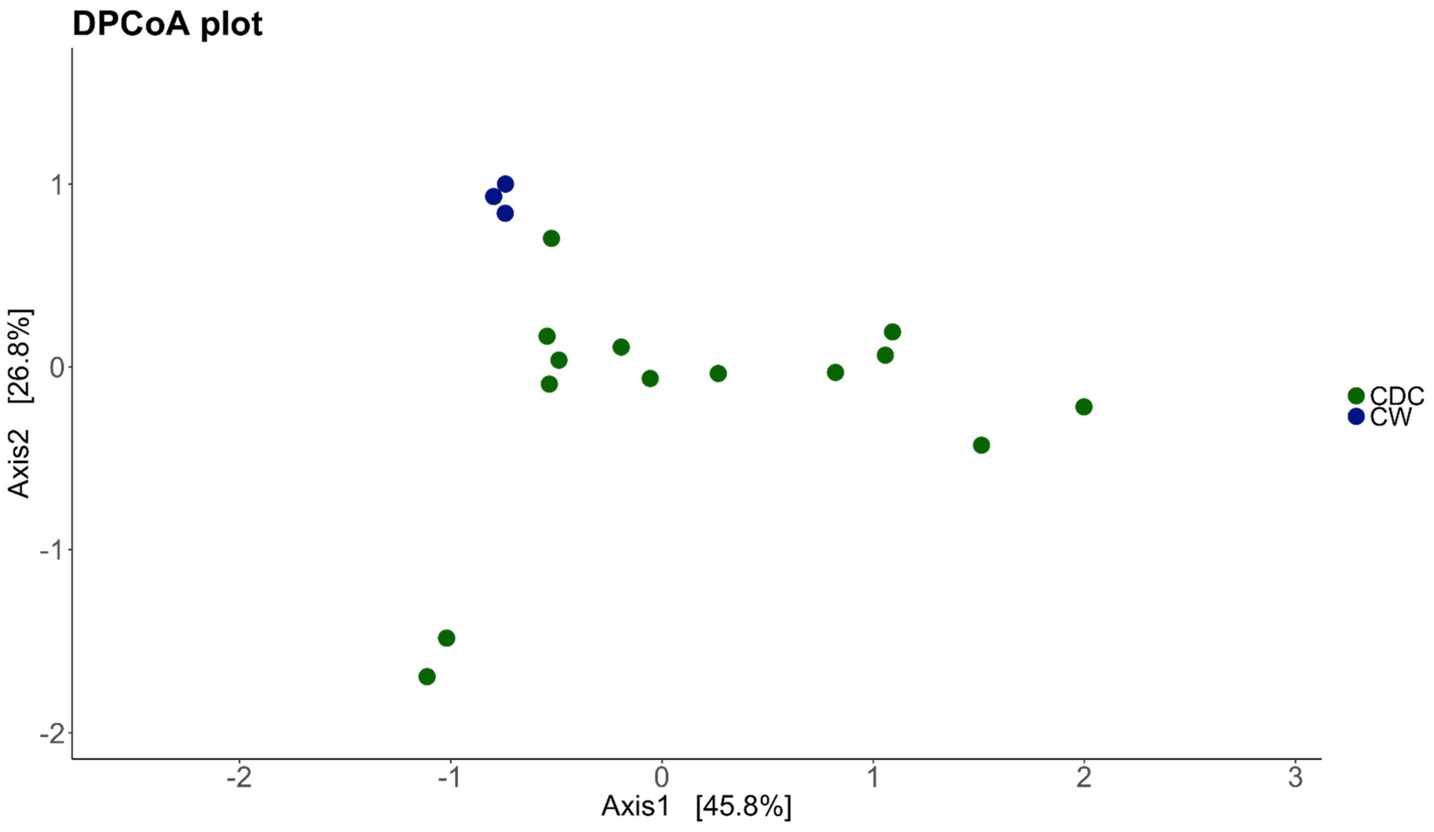

Supplementary Figure 2C

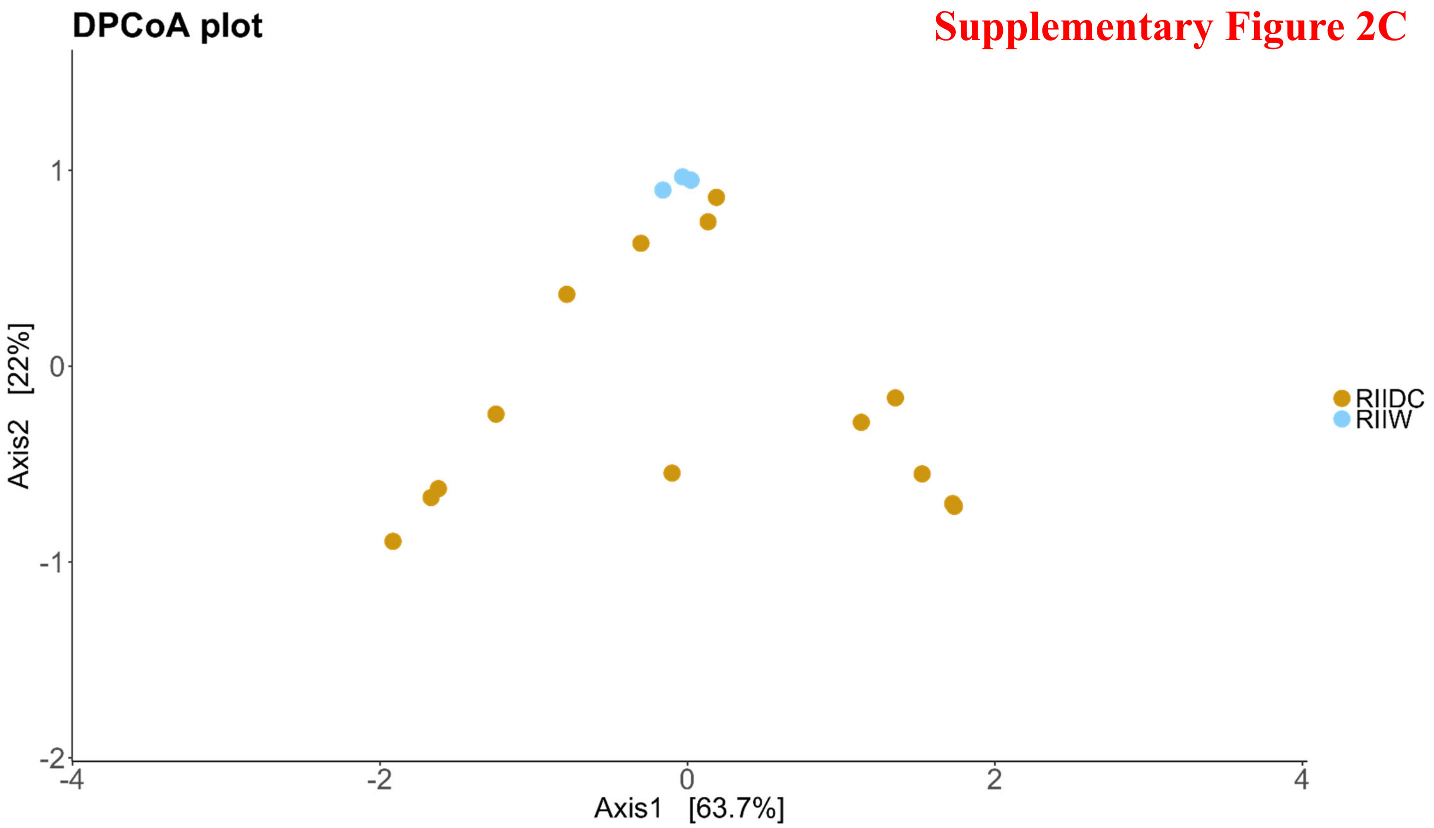


Supplementary Figure 2D

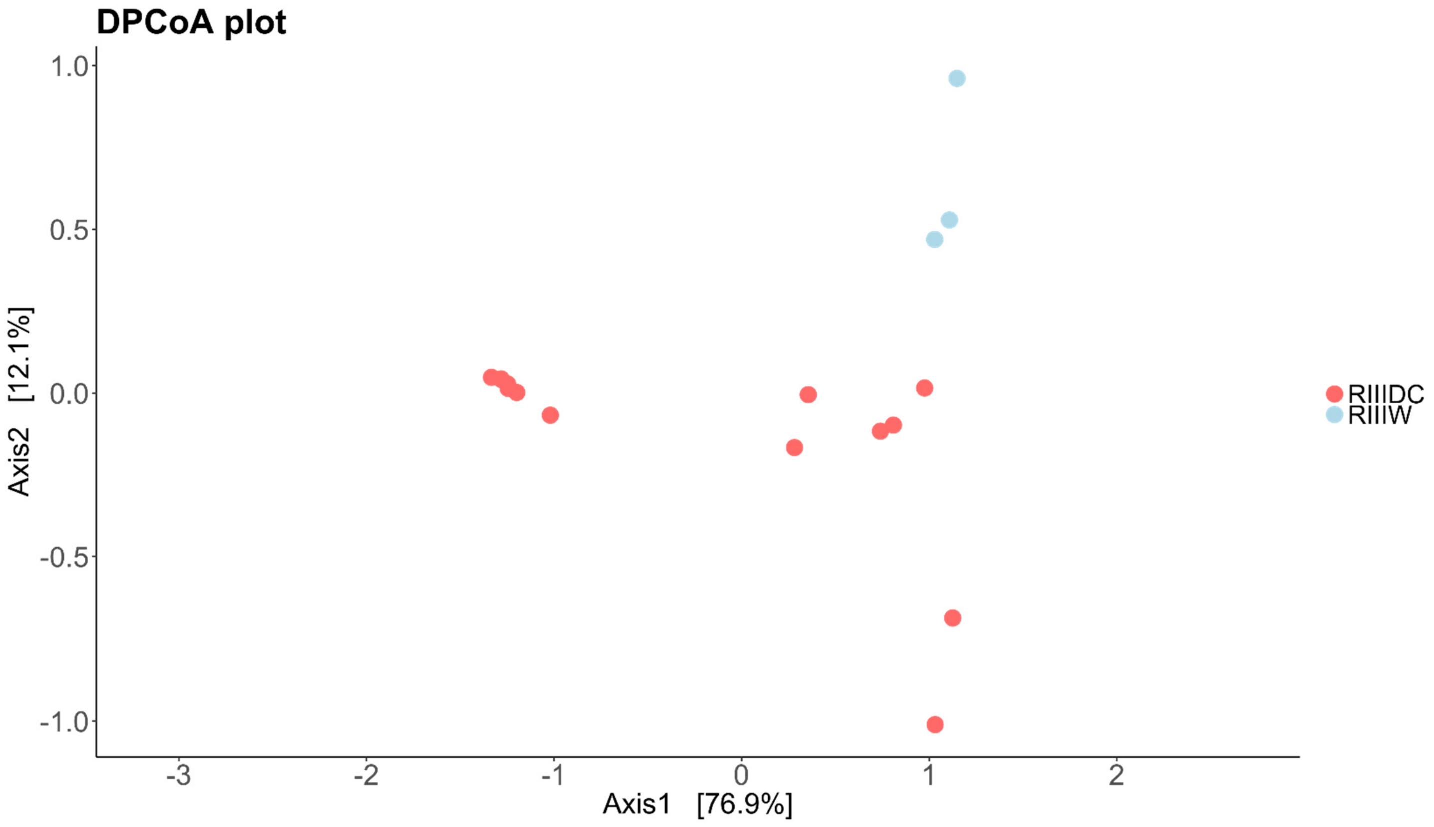

Supplementary Figure 2E

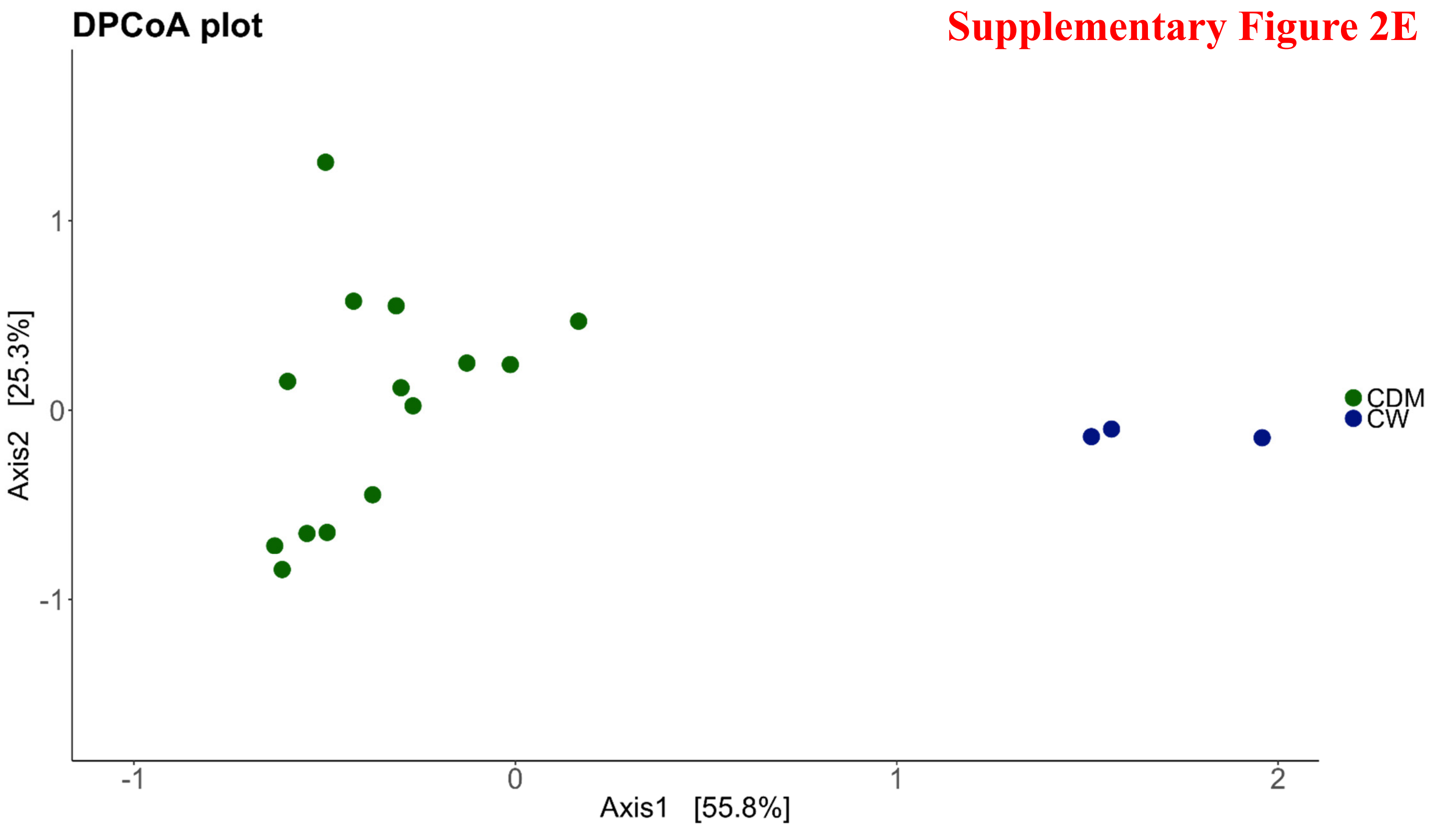


Supplementary Figure 2F

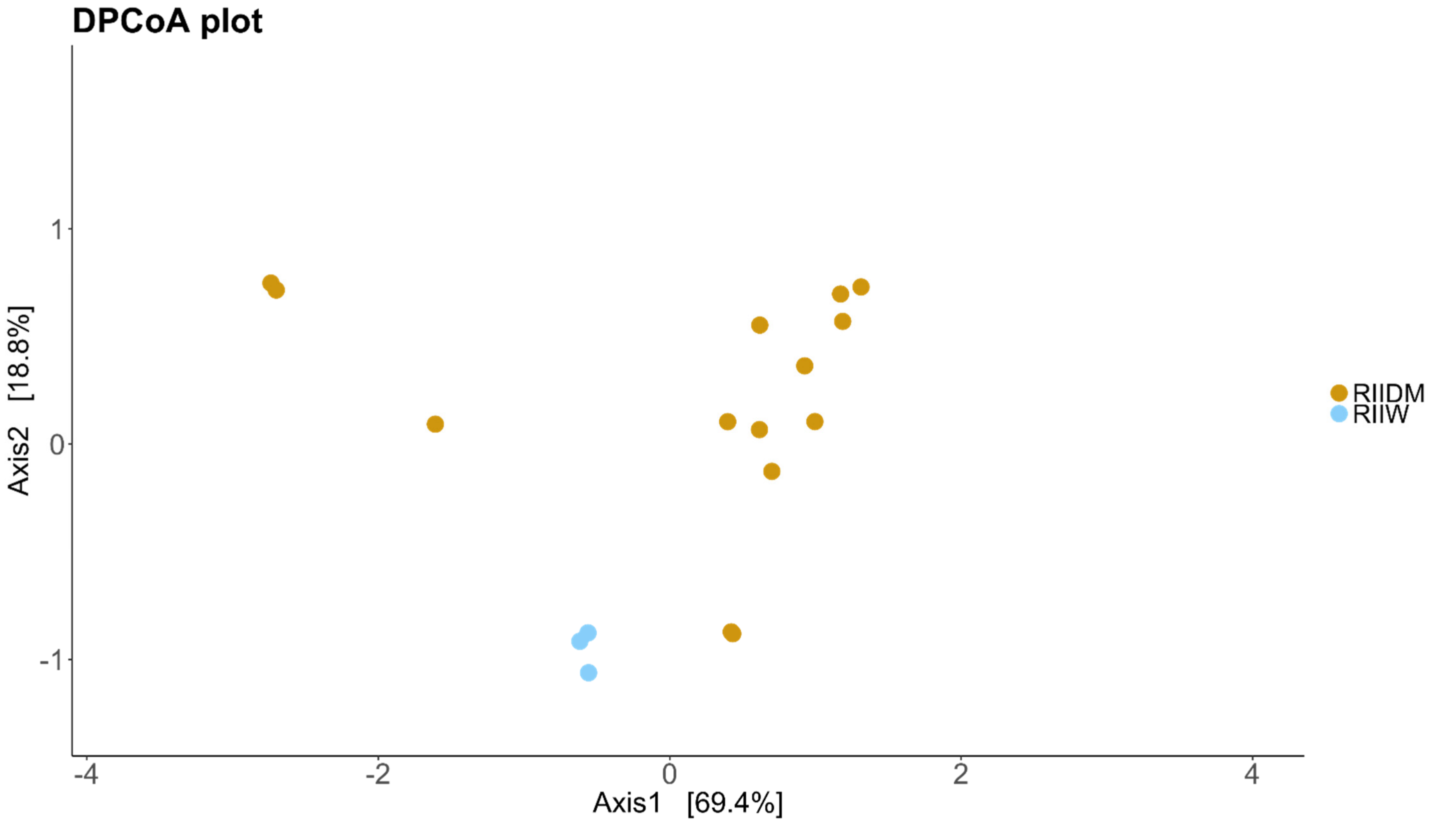

**Supplementary Figure 2G**

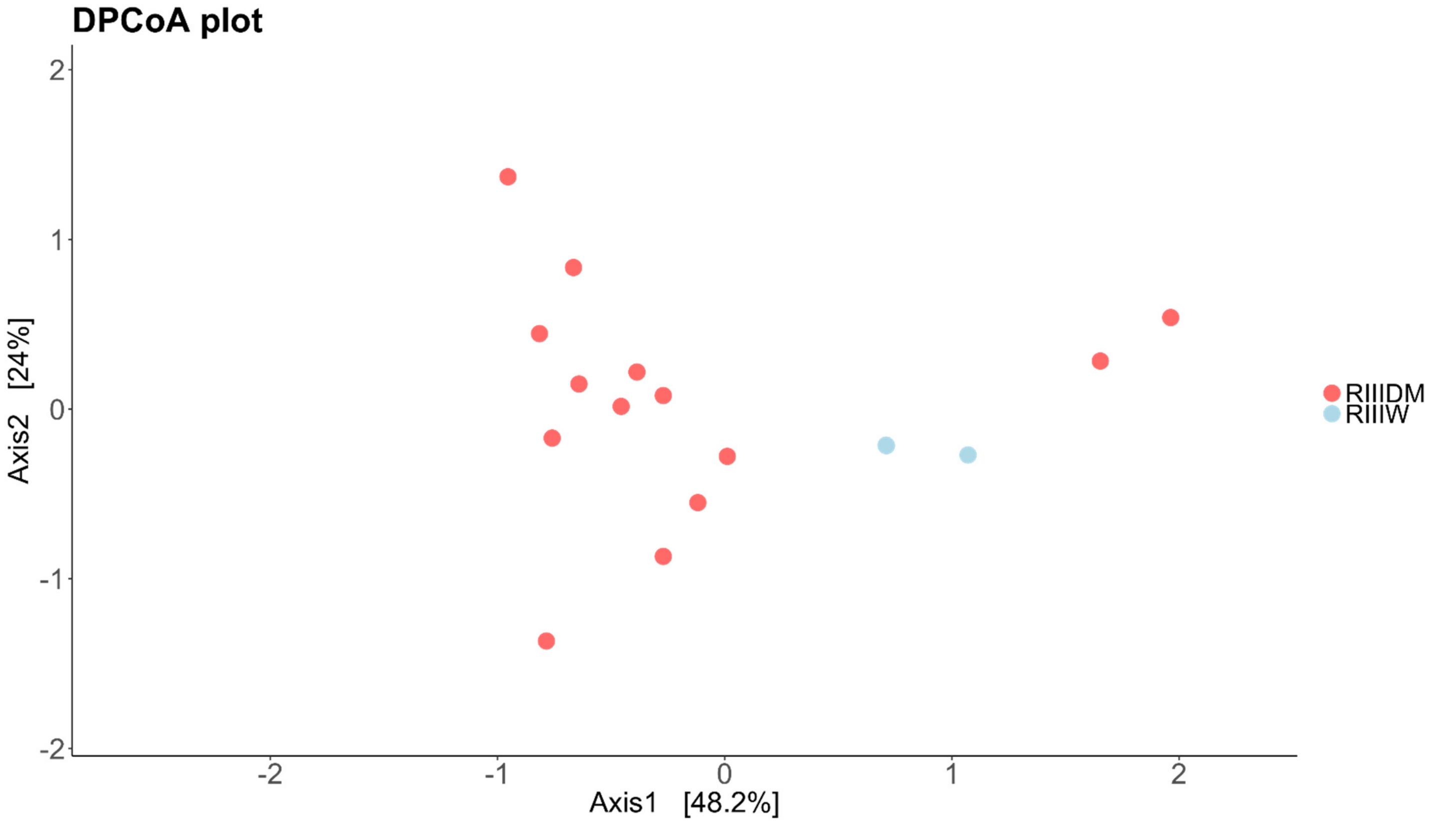

**Supplementary Figure 3A**

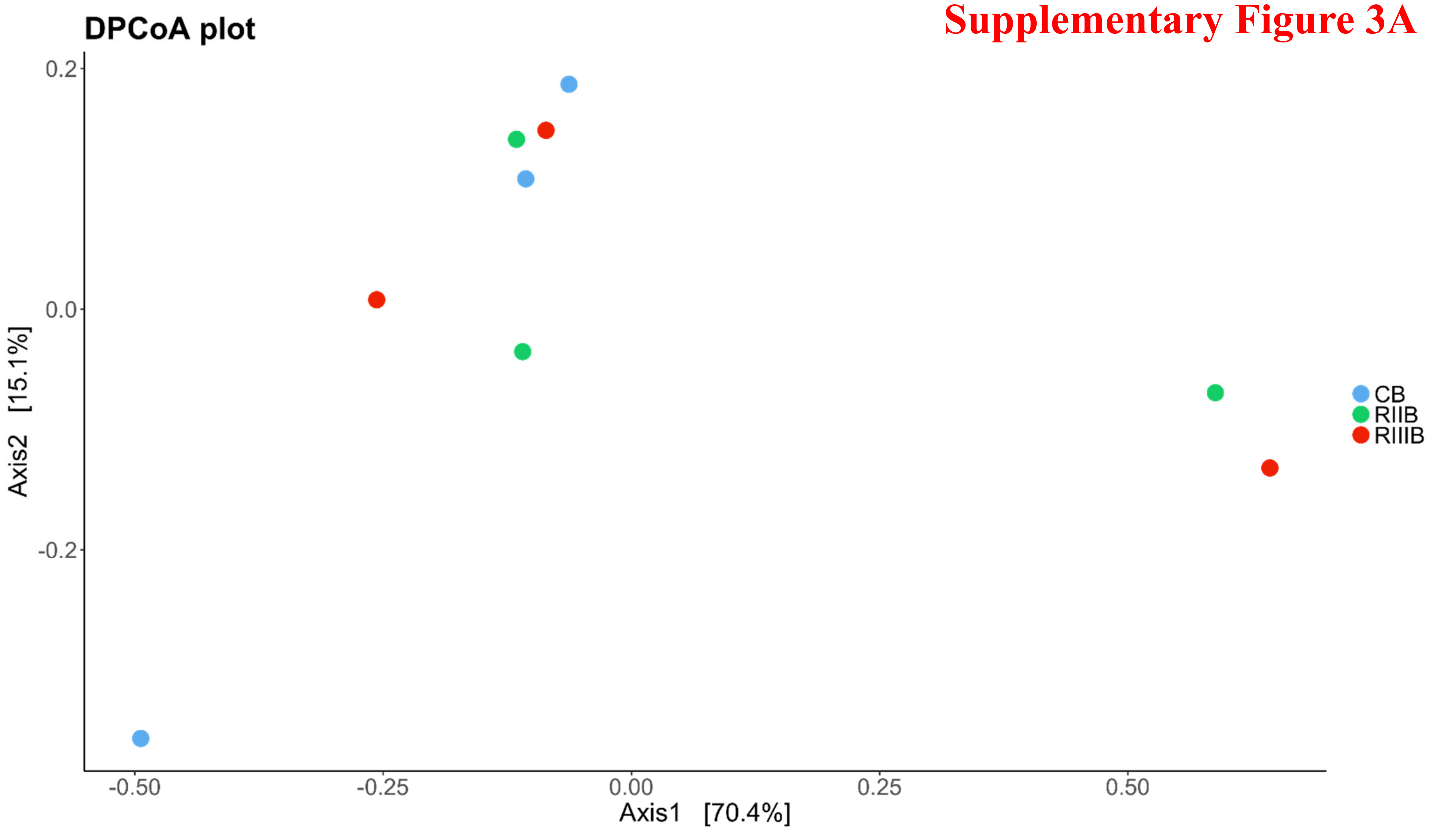


**Supplementary Figure 3B**

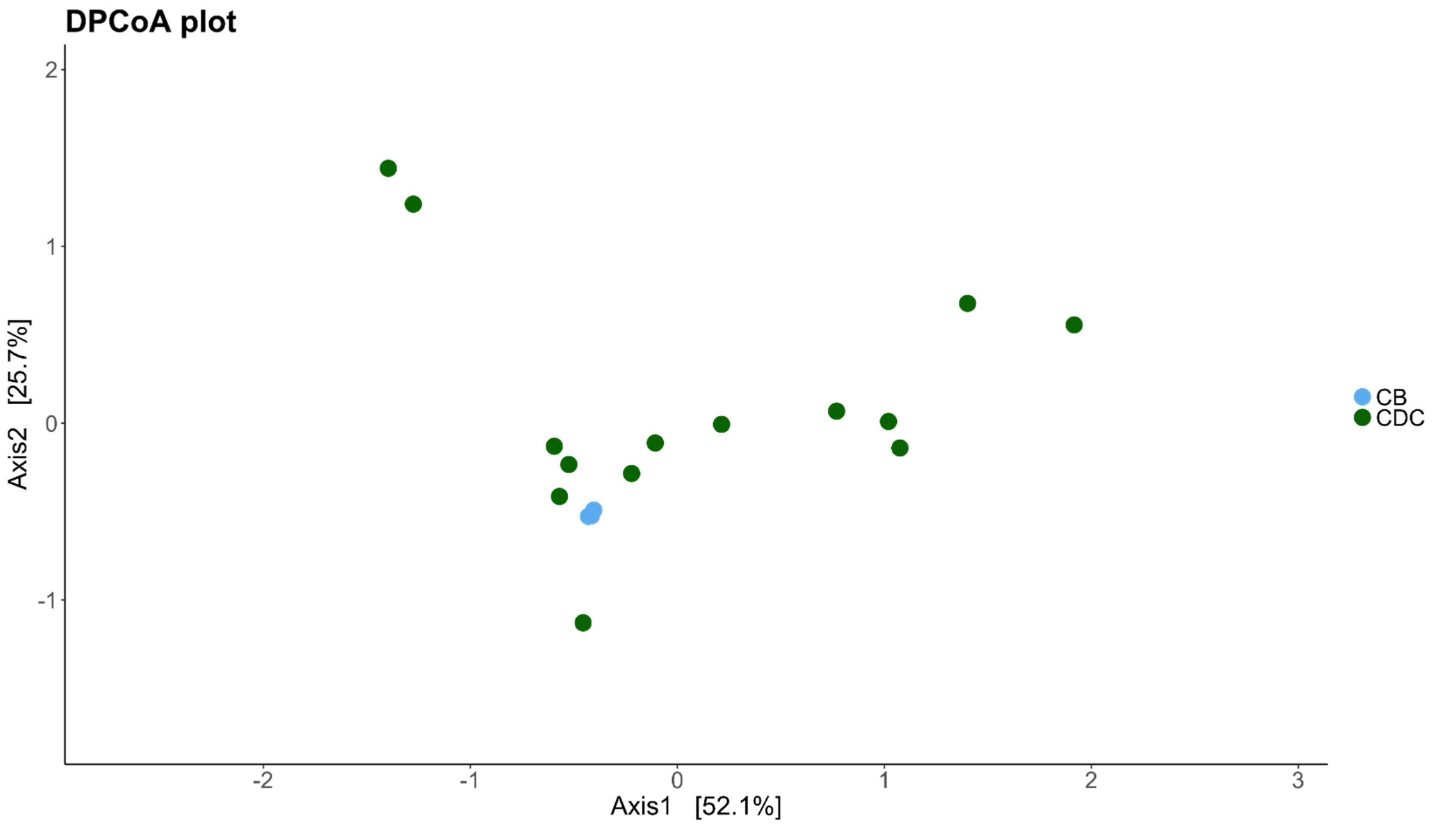

Supplementary Figure 3C

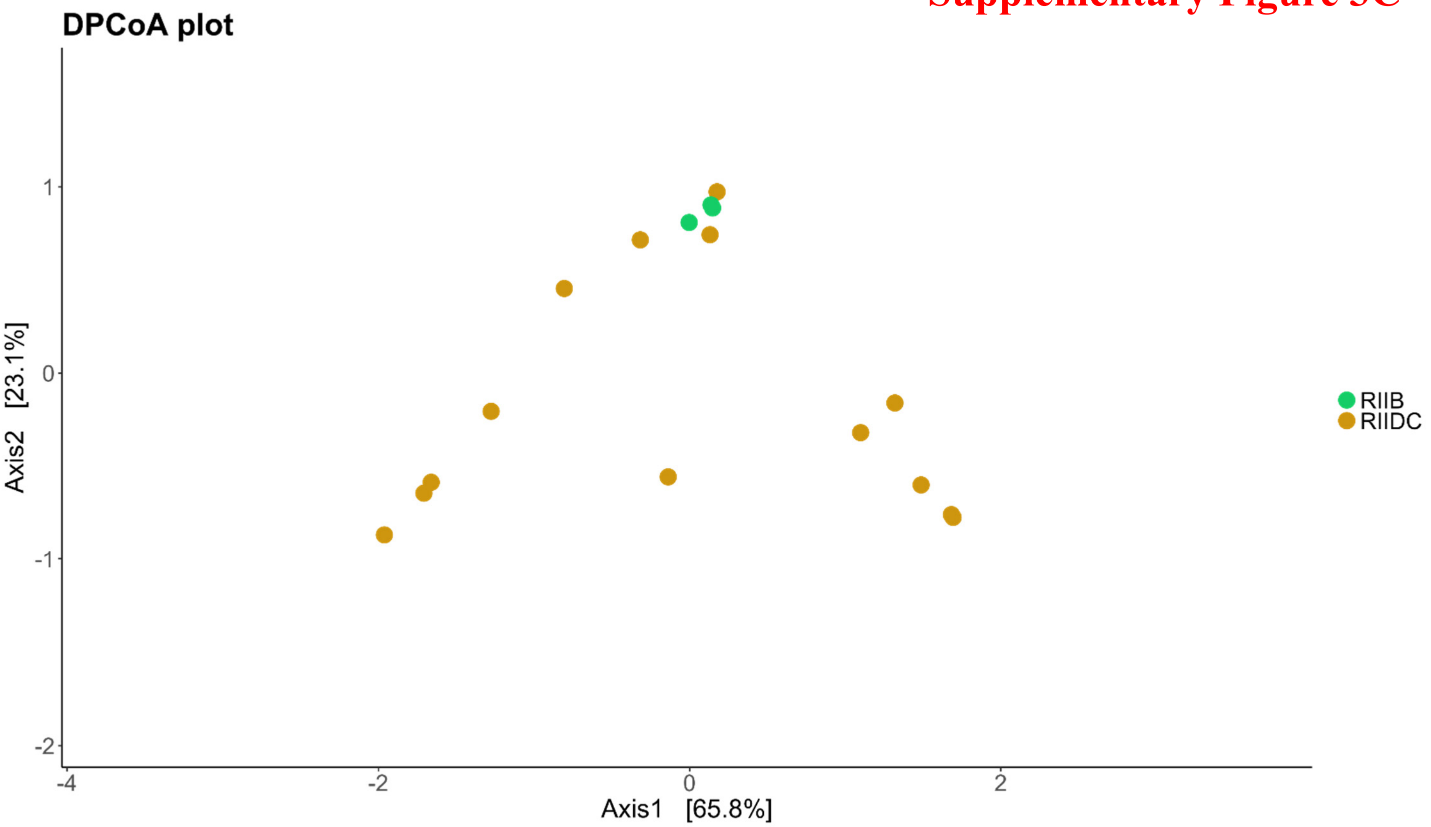


Supplementary Figure 3D

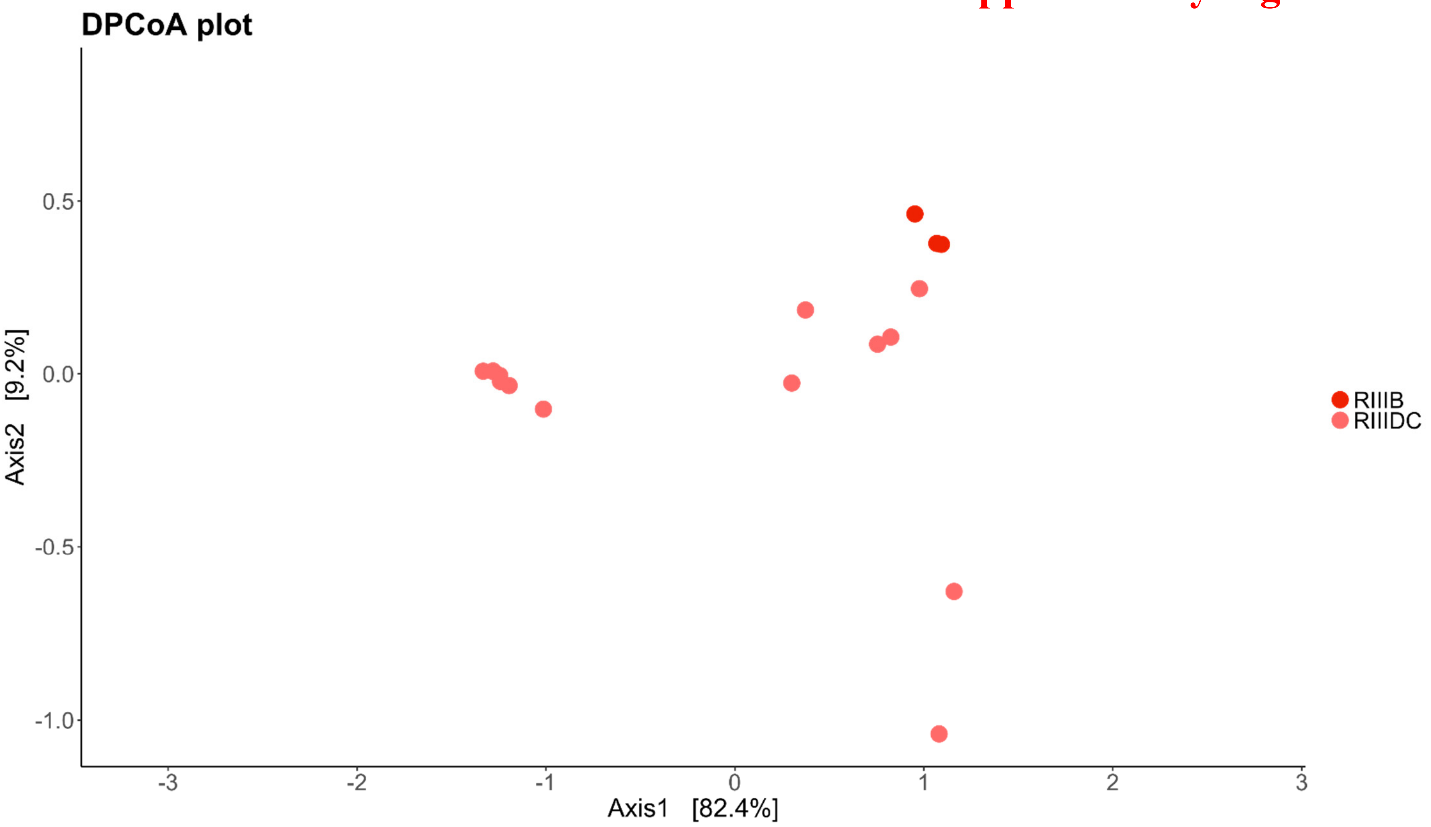

## Supplementary Figure 3E

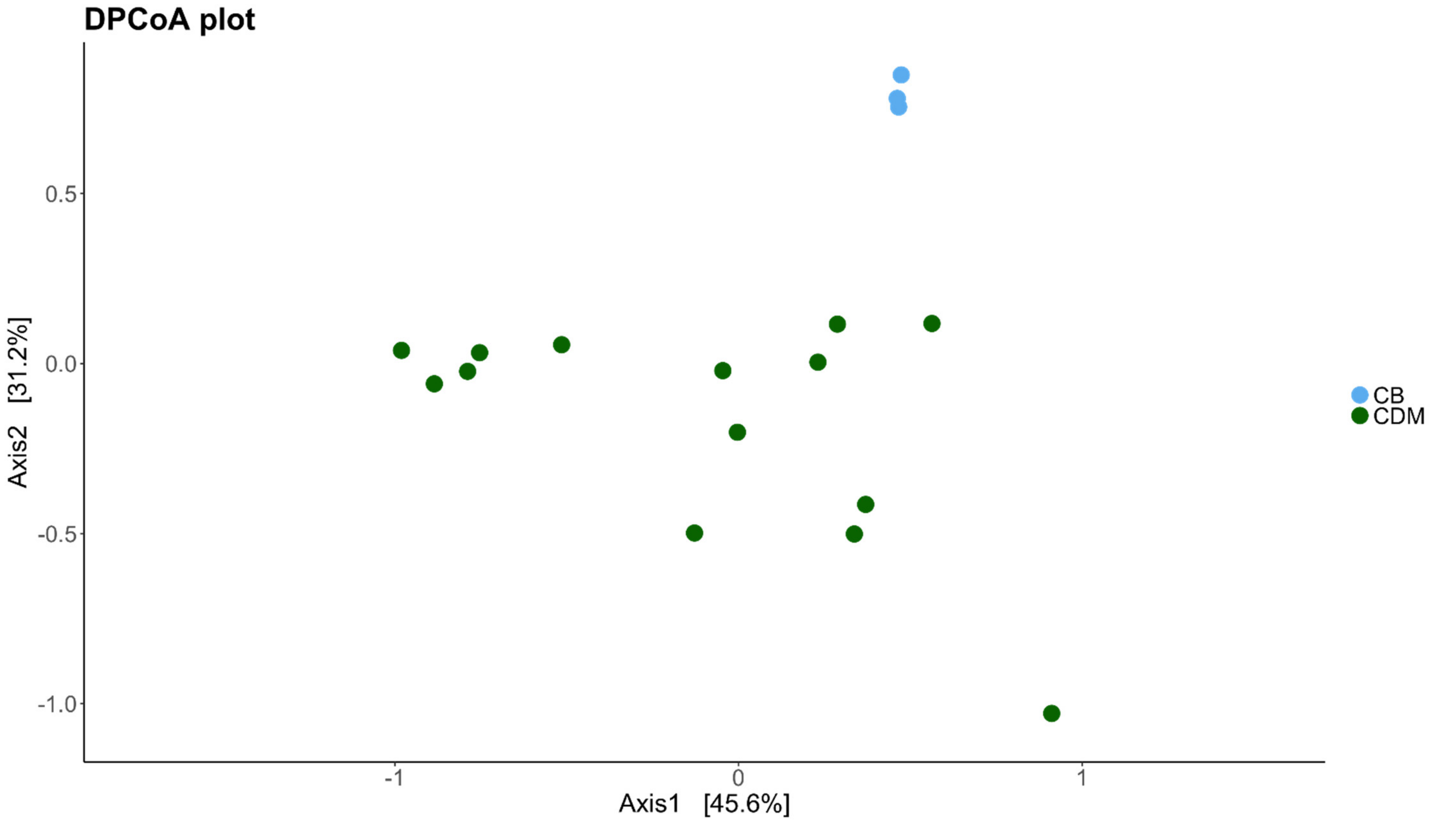


## Supplementary Figure 3F

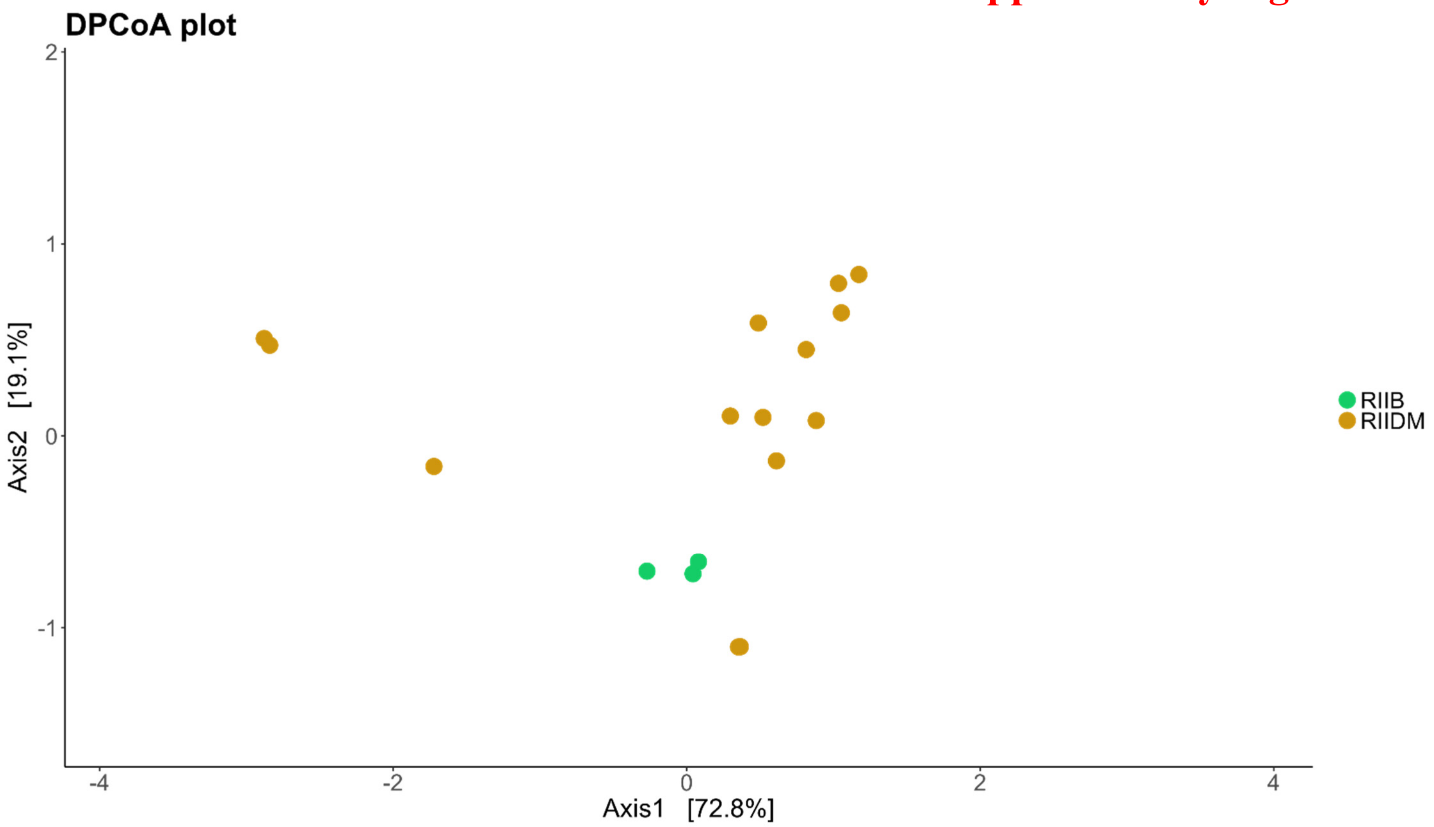

**Supplementary Figure 3G**

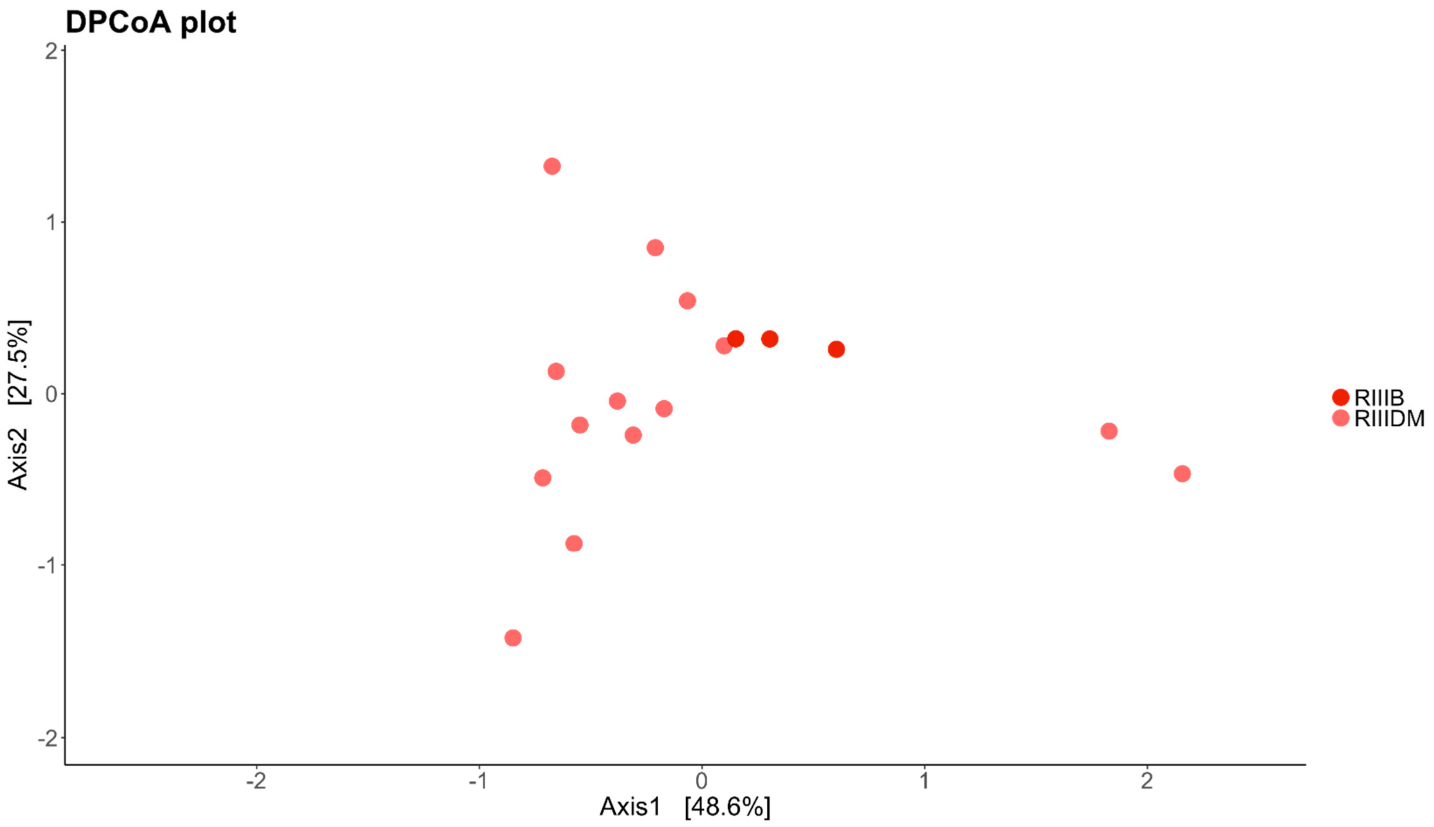

**Supplementary Figure 4A**

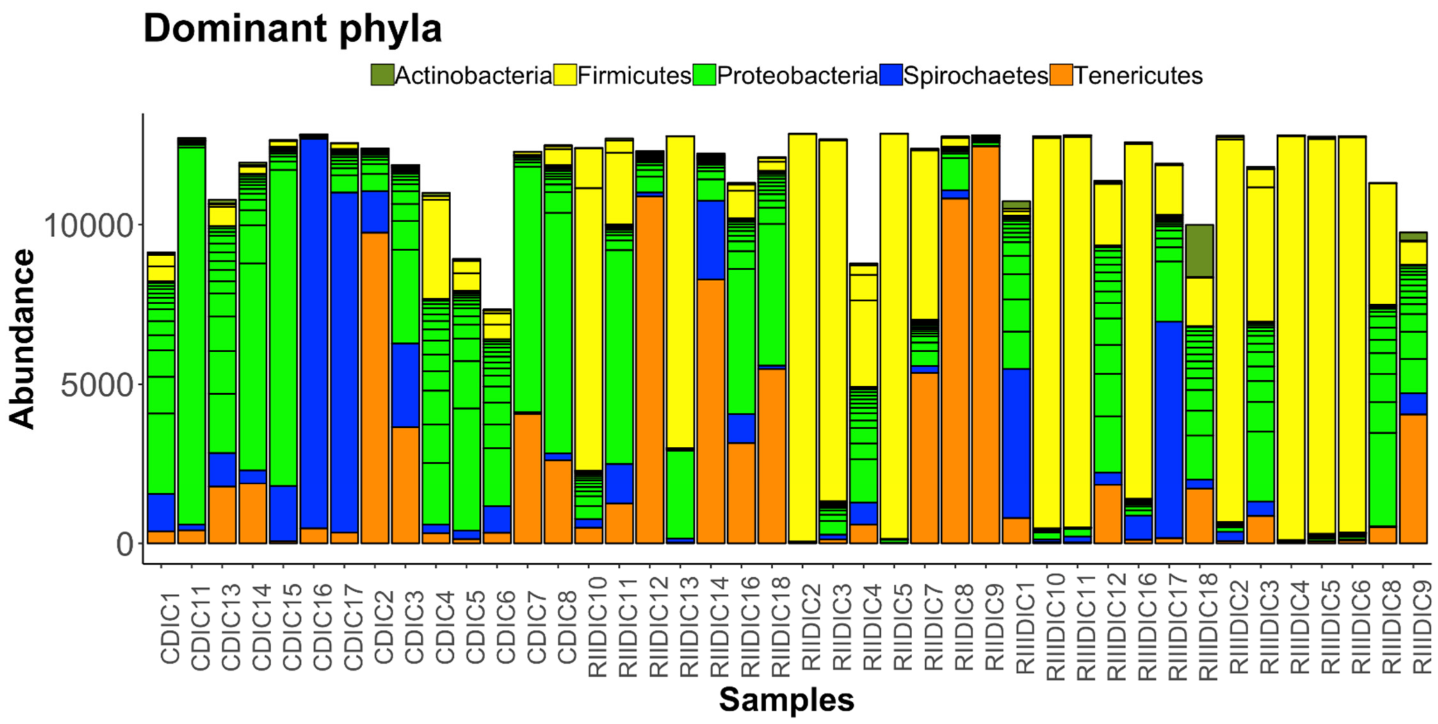


**Supplementary Figure 4B**

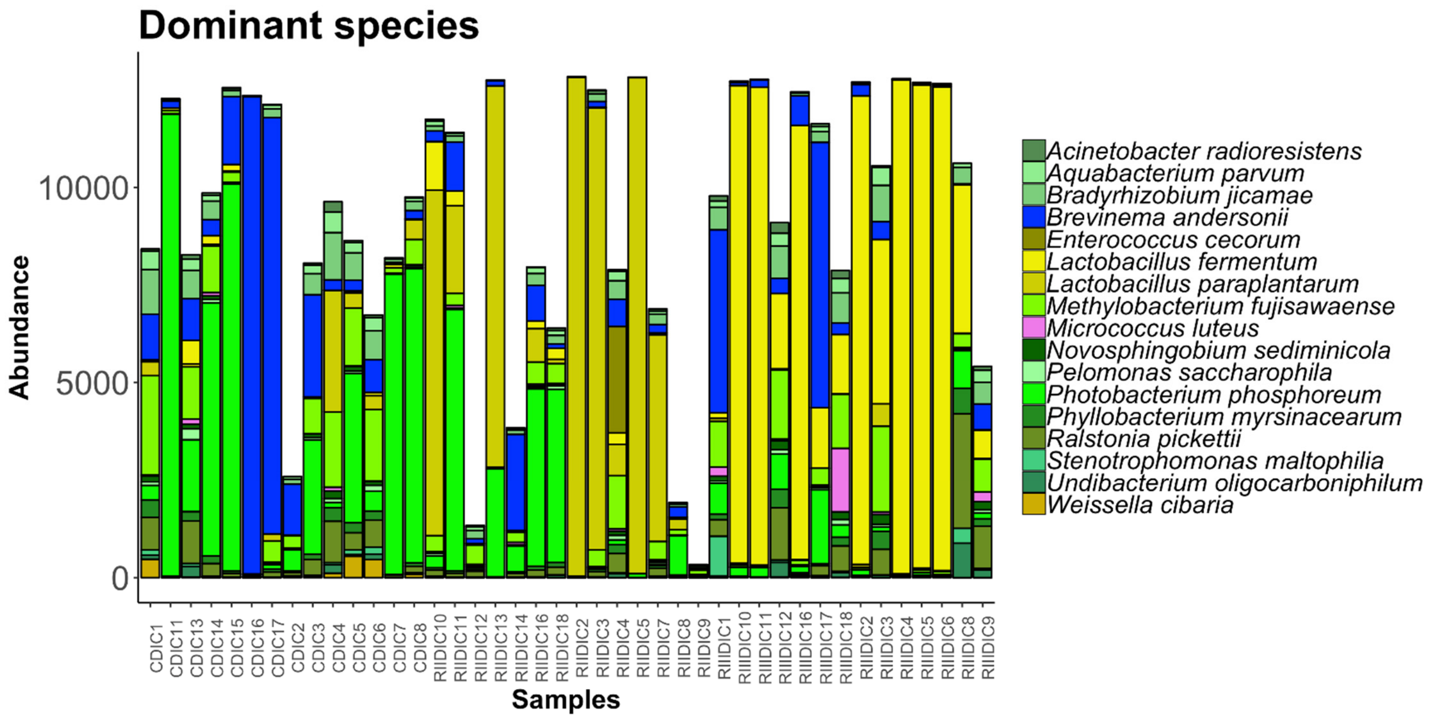

**Supplementary Figure 4C**

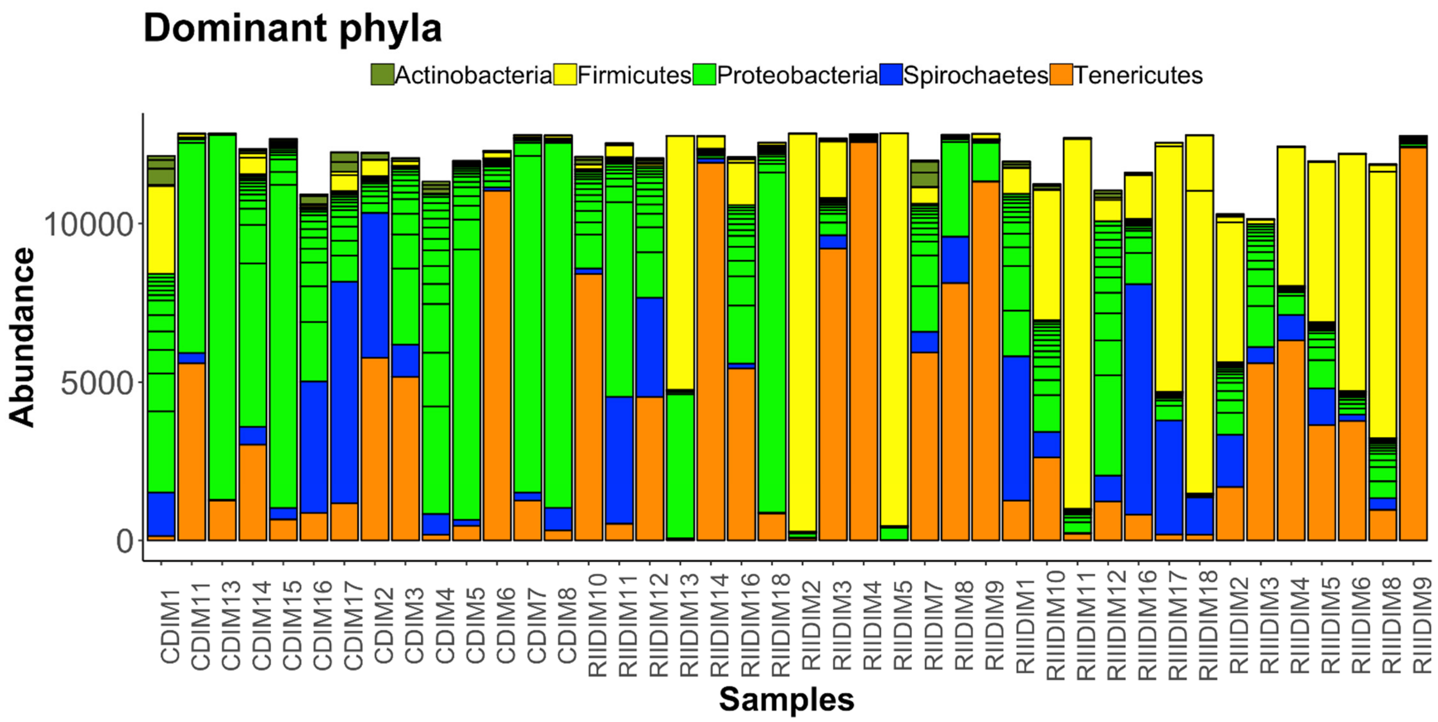


**Supplementary Figure 4D**

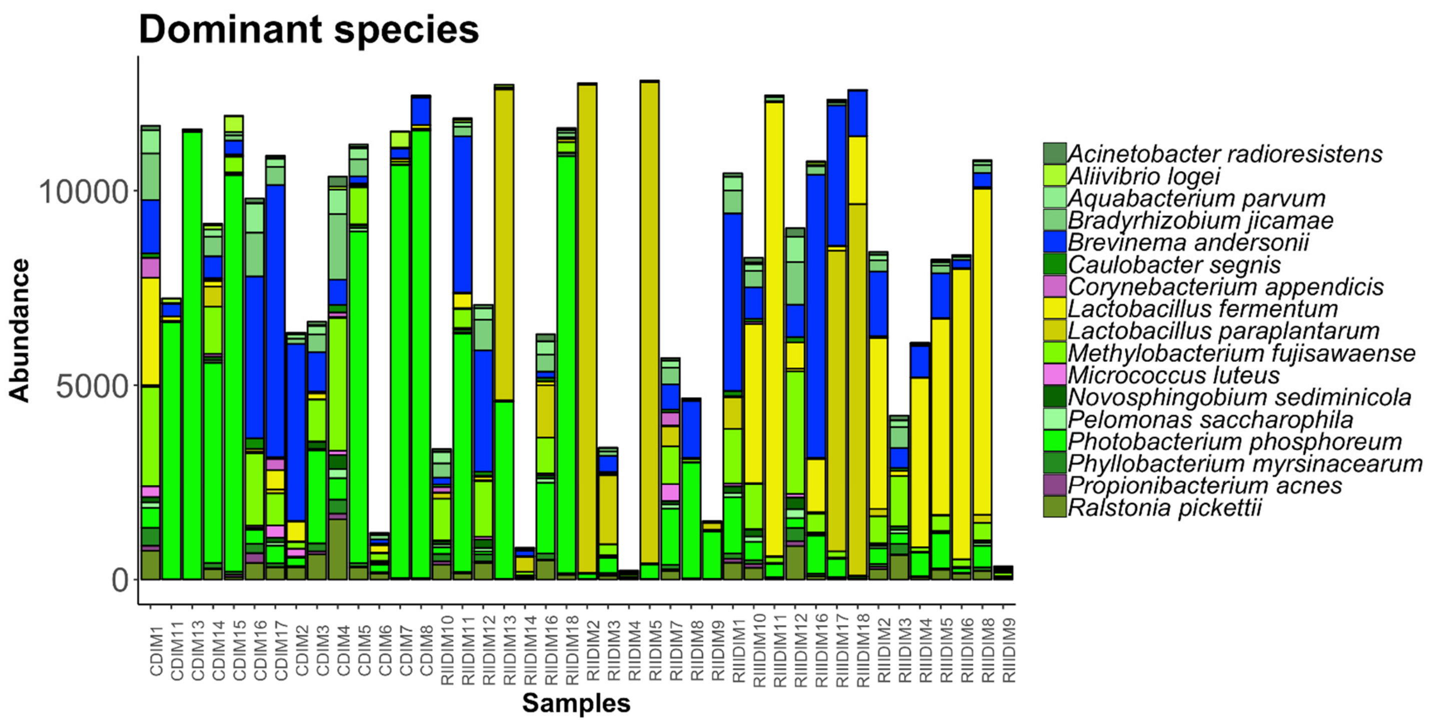

## Supplementary Figure 5A

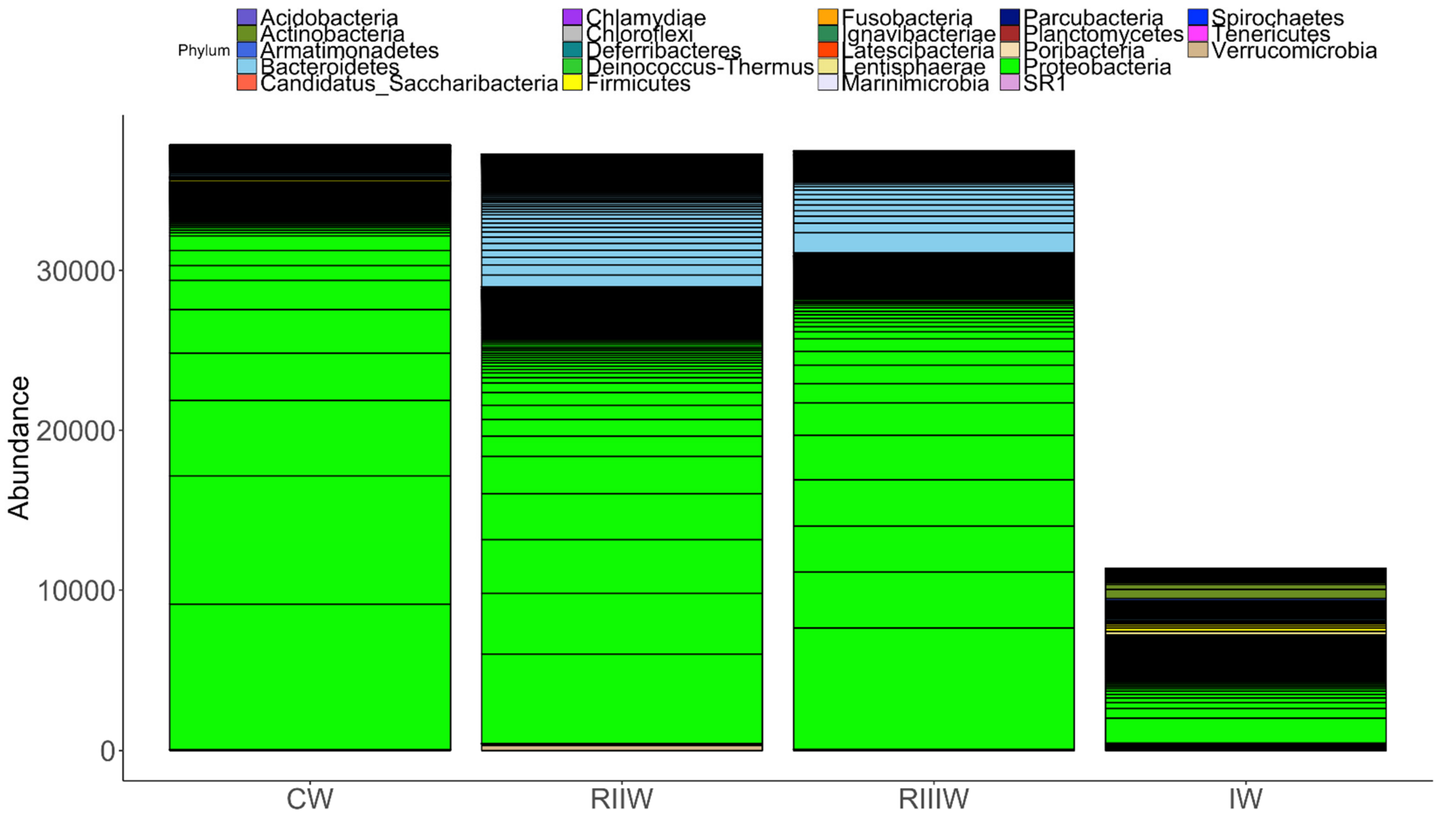


## Supplementary Figure 5B

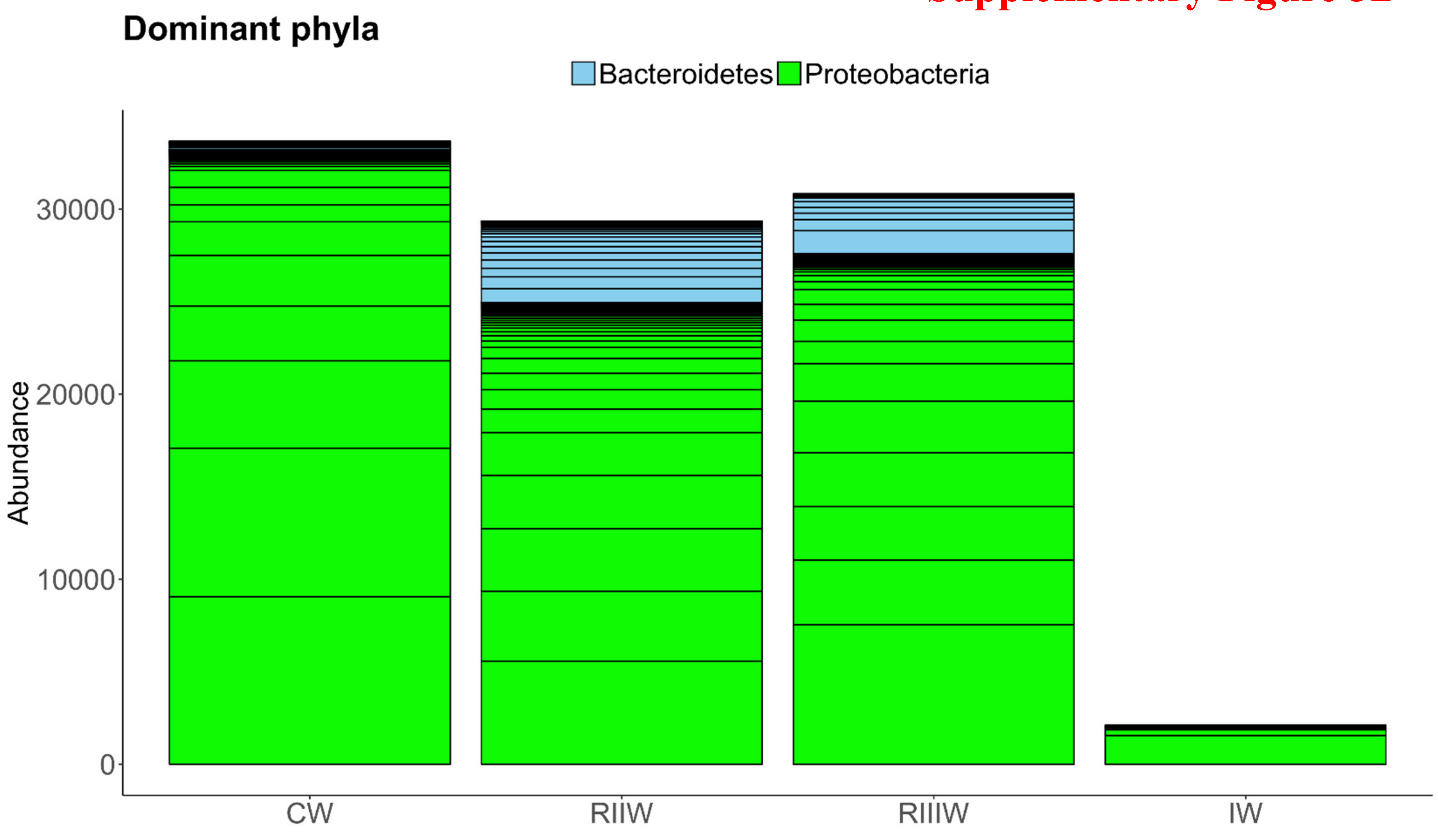

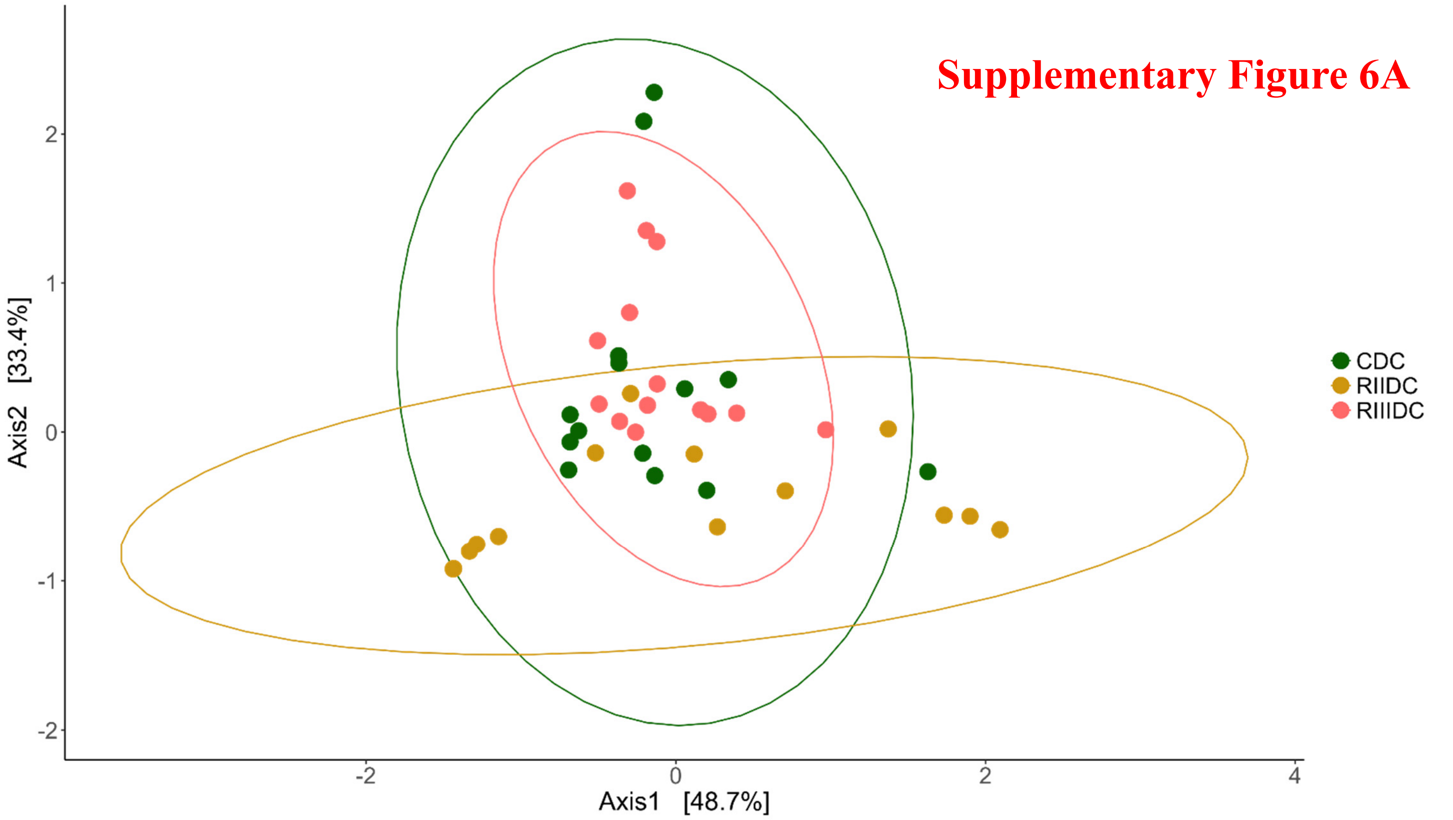
Supplementary Figure 6A
CDC
RIIDC
RIIIDC
Axis2 [33.4%]
Axis1 [48.7%]
2
1
0
-1
-2
-2
0
2
4

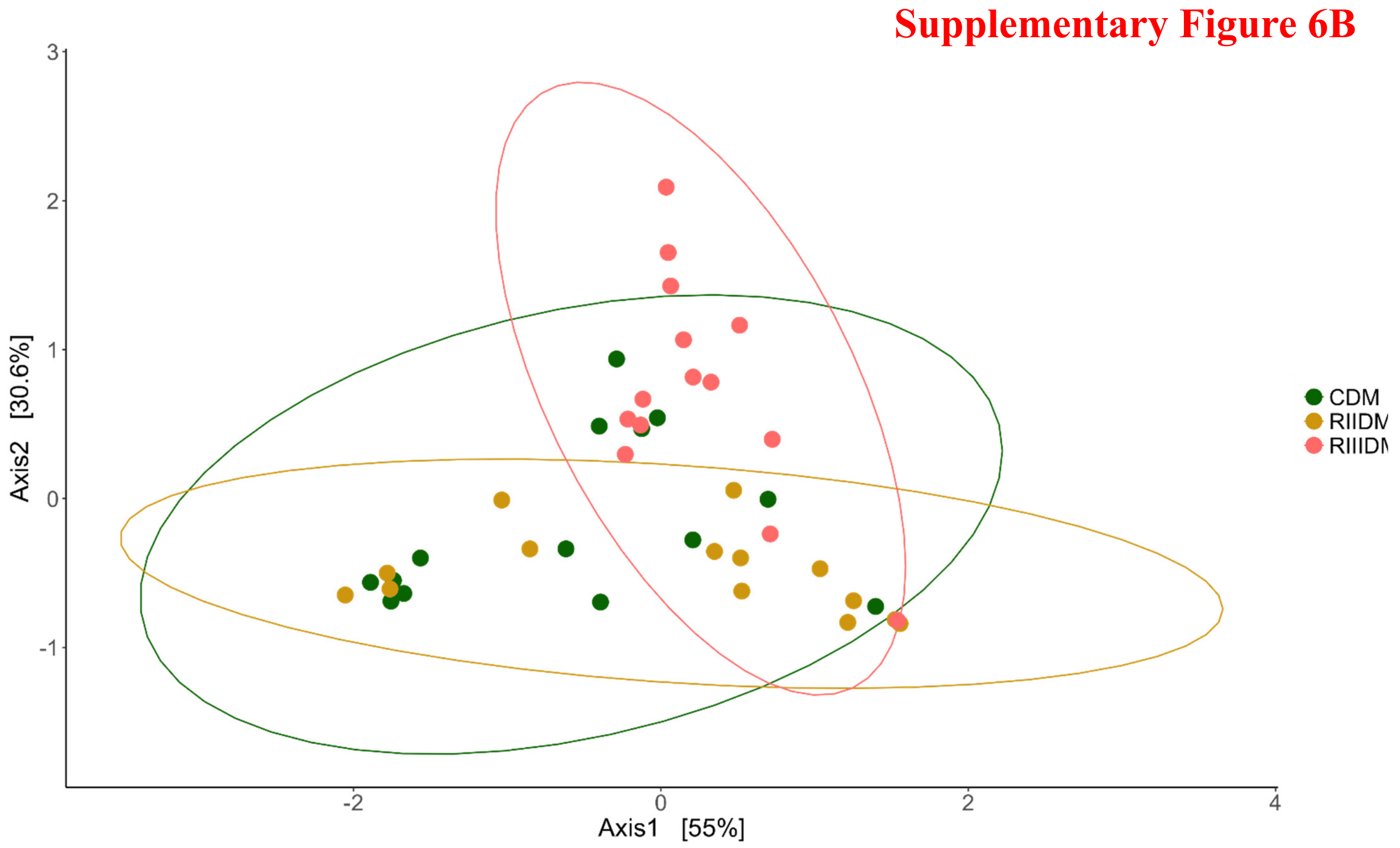
Supplementary Figure 6B
CDM
Axis2 [30.6%]
Axis1 [55%]
3
2
1
0
-1
-2
0
2
4

# Supplementary Figure 7

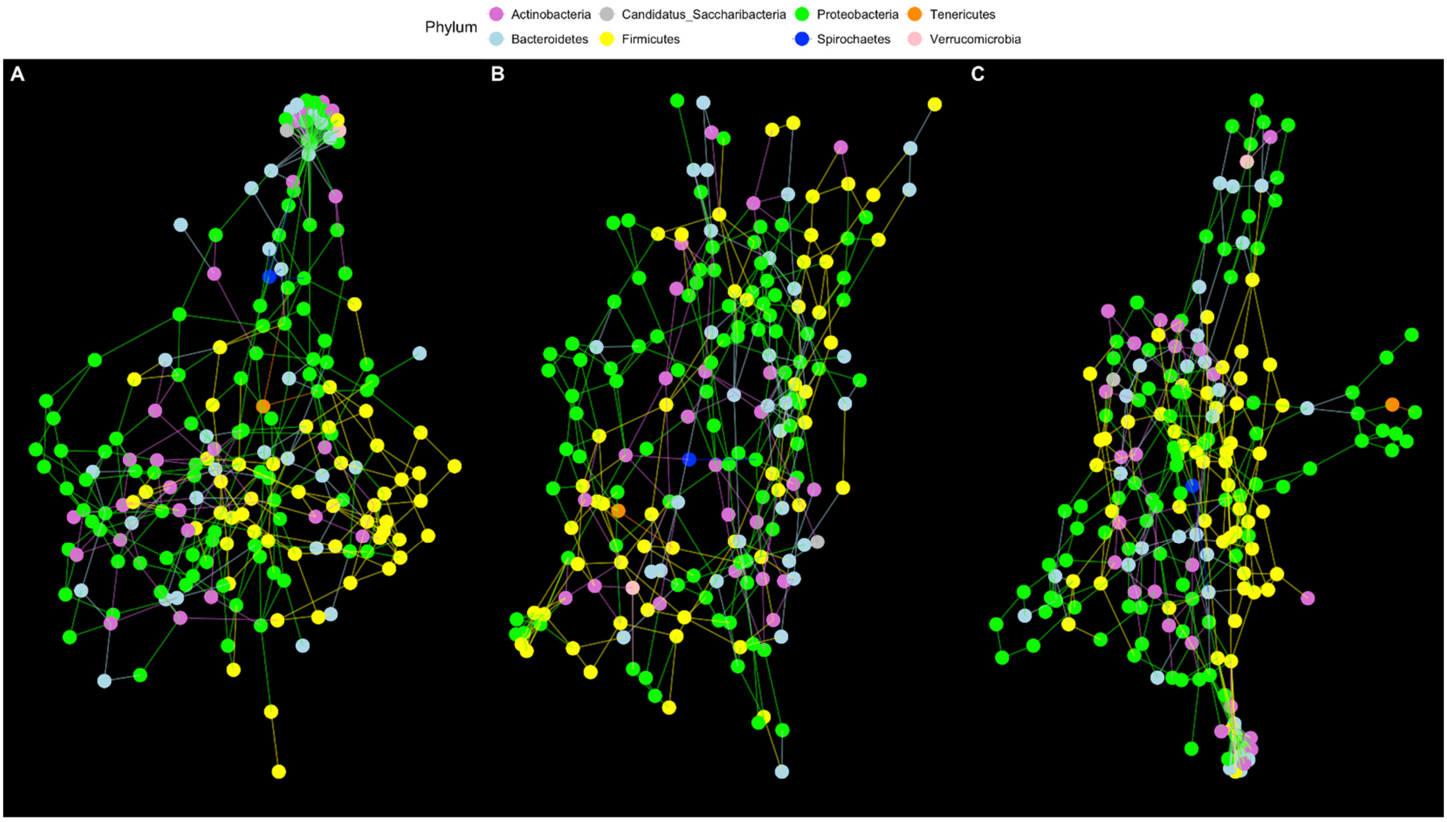


# Supplementary Figure 8

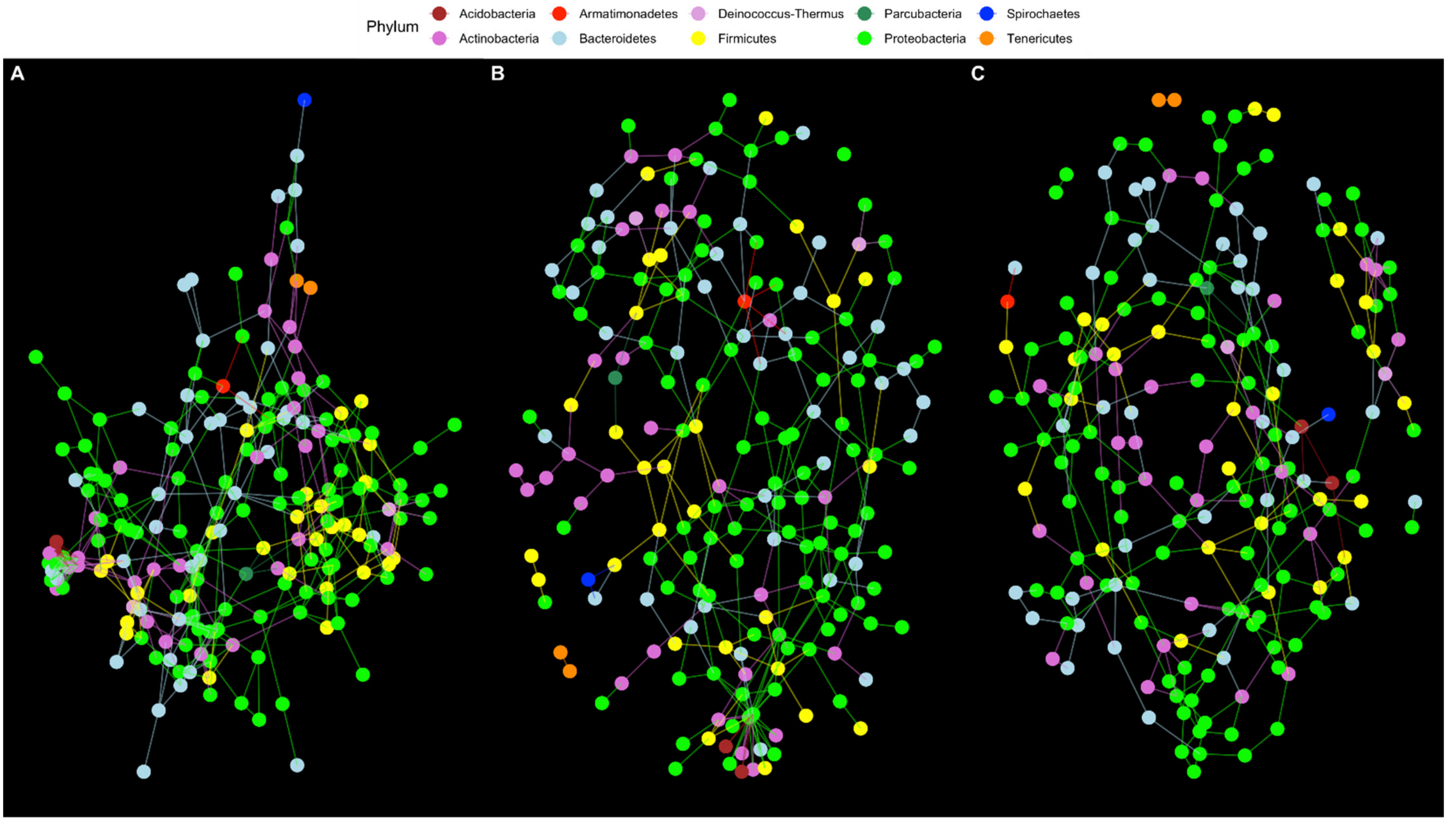

## Supplementary Figure 9A

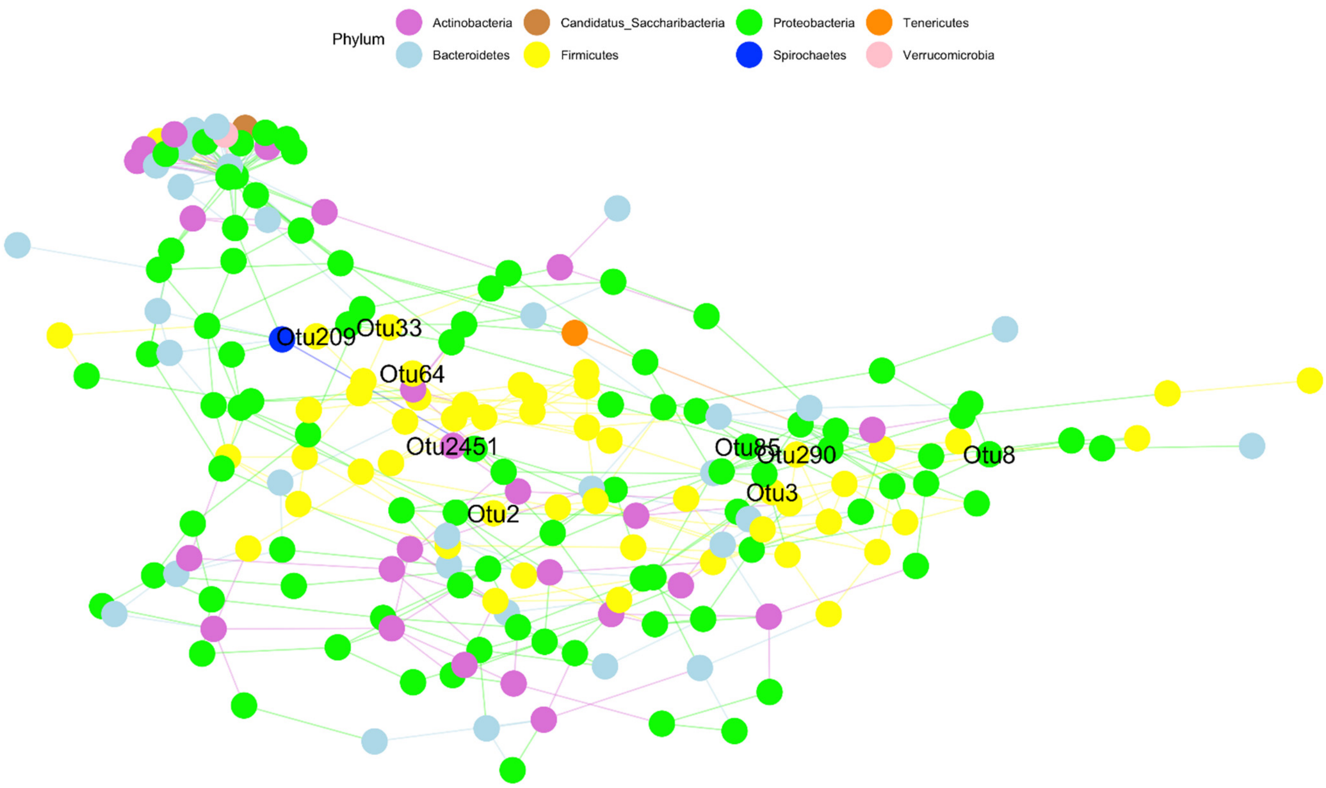


## Supplementary Figure 9B

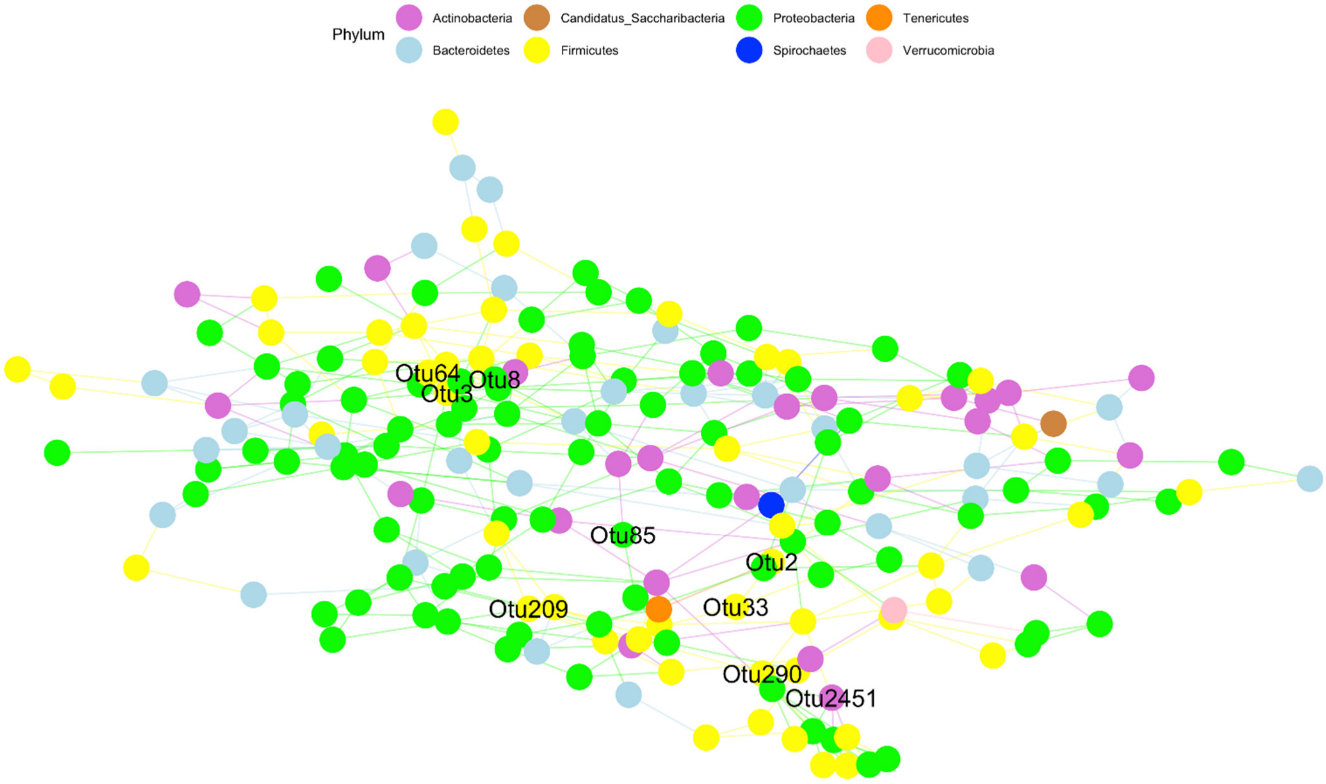

# Supplementary Figure 9C

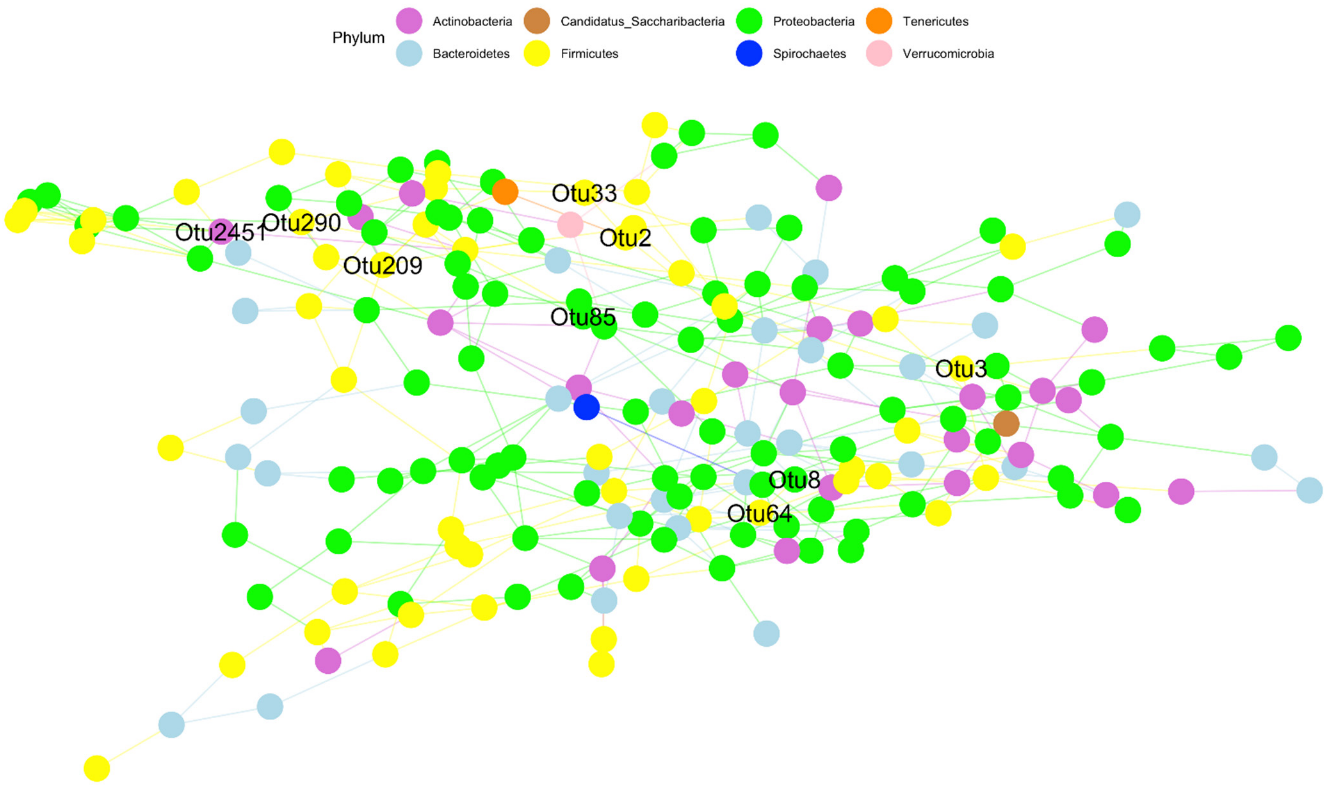

## Supplementary Figure 10A

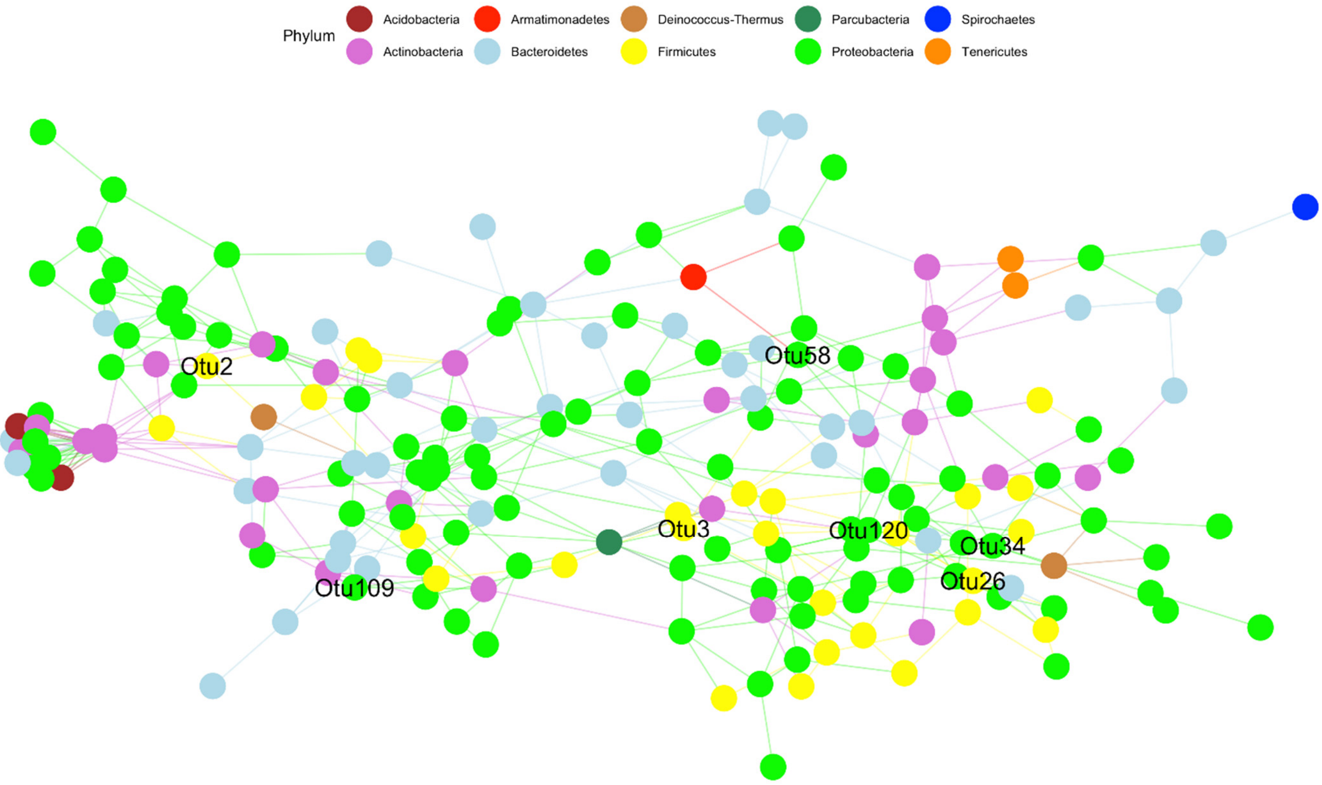


## Supplementary Figure 10B

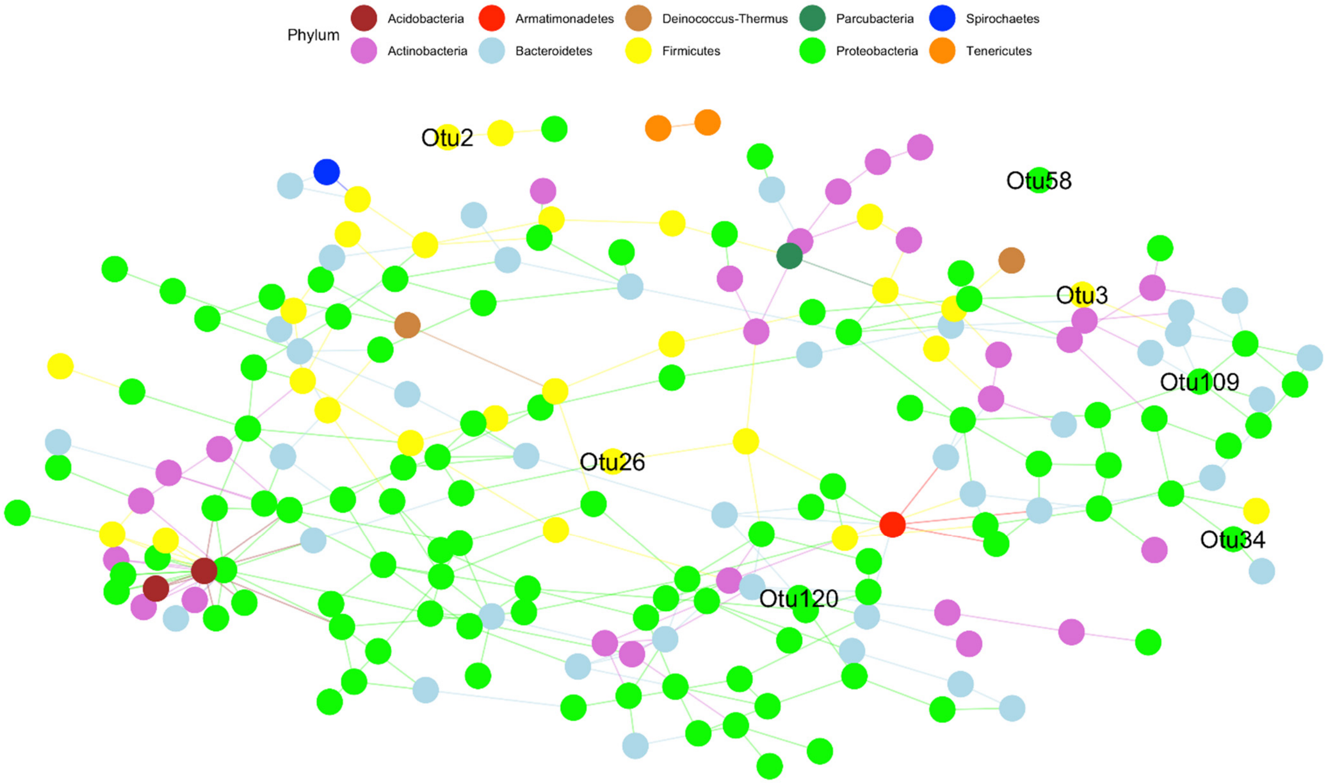

# Supplementary Figure 10C

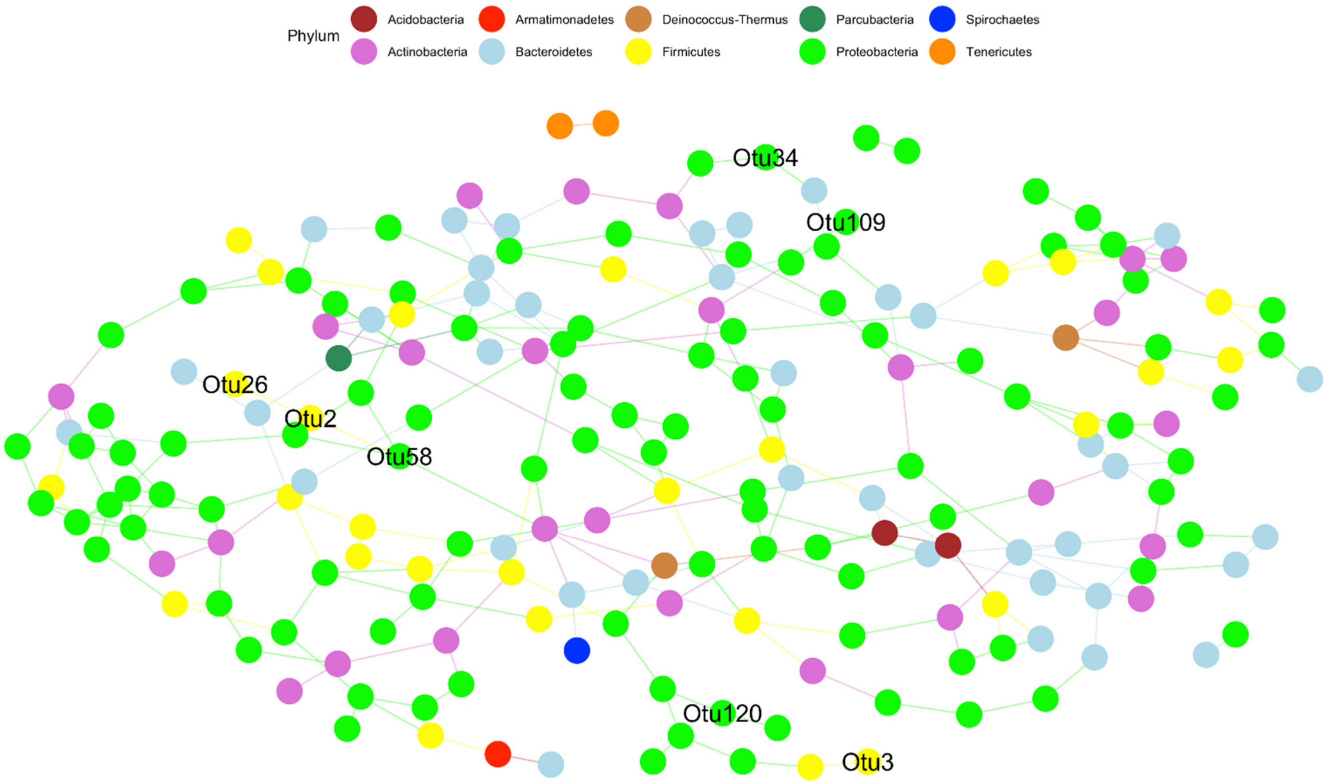

**Supplementary Figure 11A**

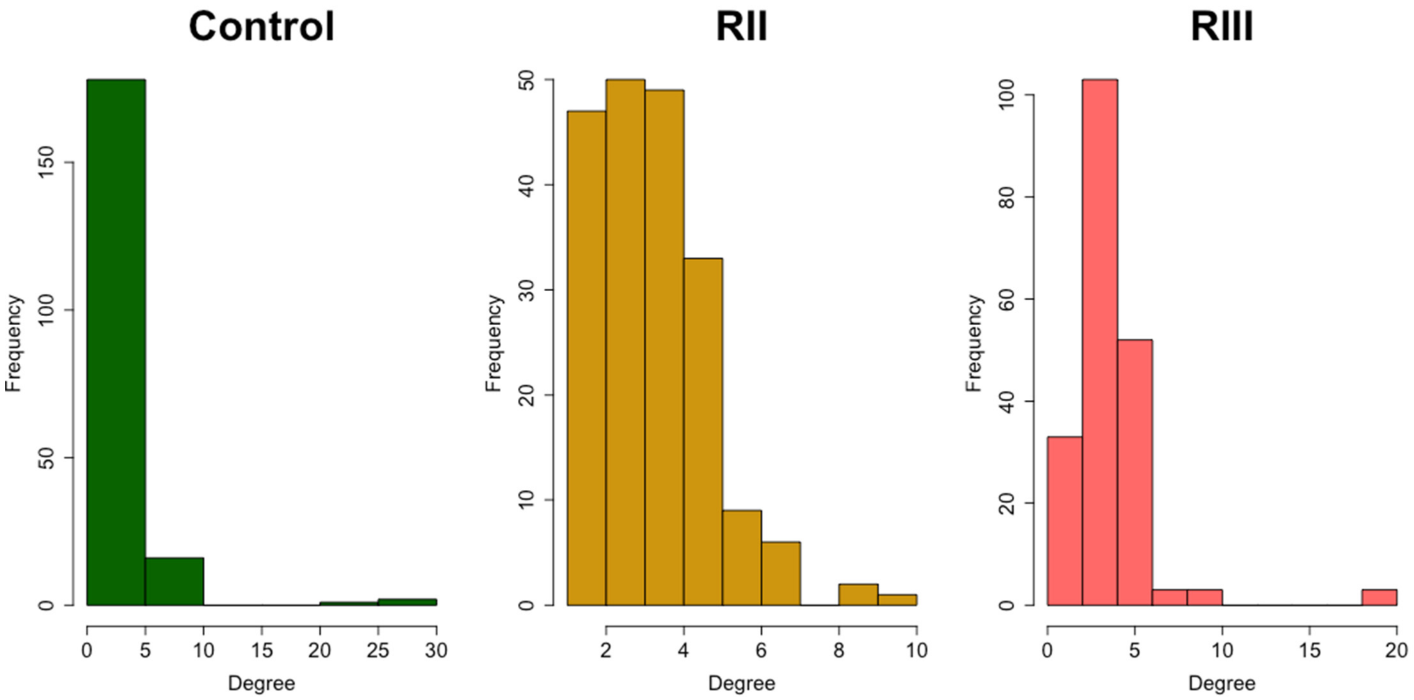


**Supplementary Figure 11B**

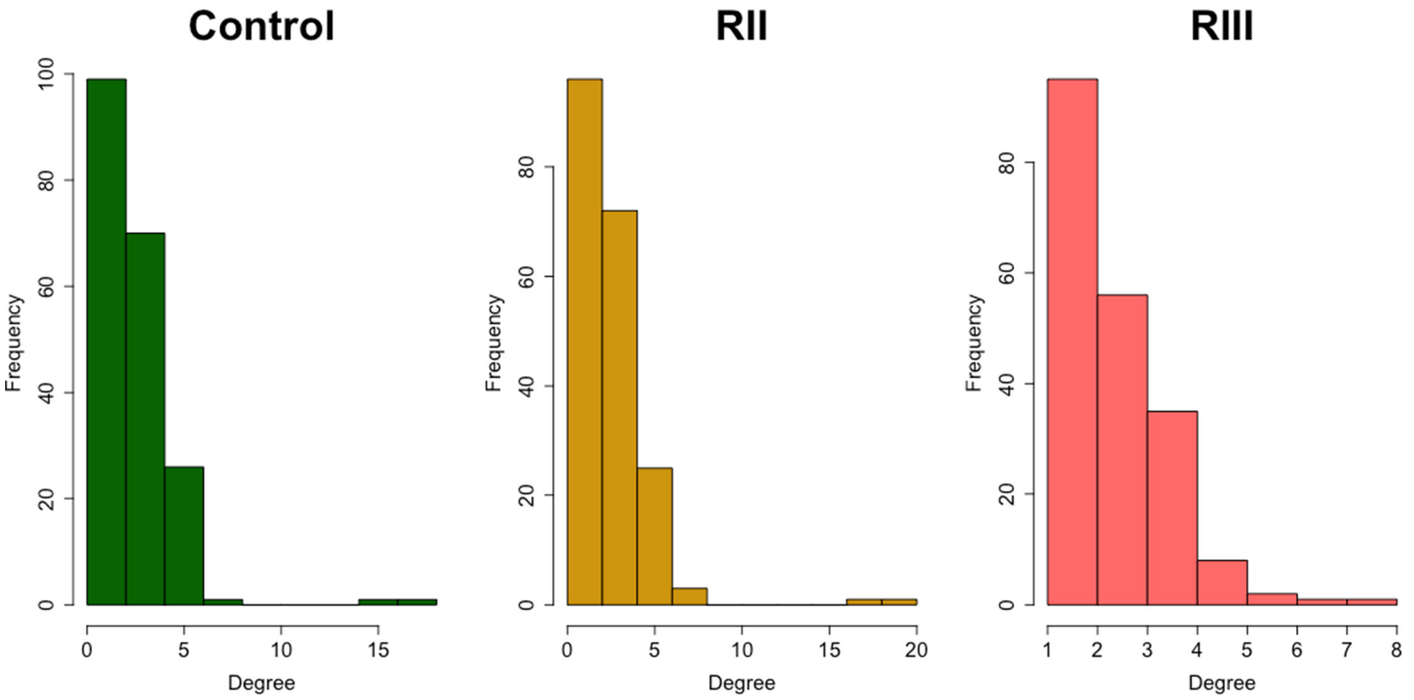

Supplement: Supplementary Figure 1 — Sample-size-based rarefaction curves for the reads obtained from the intestinal content (A) and mucus (B). The shaded portion around each line represents the 95% confidence interval. Color code for the feed groups: green lines- control, orange lines- RII, pink lines- RIII. Codes for content samples: CDM-control, RIIDC-RII, RIIIDC-RIII. Codes for mucus samples: CDM-control, RIIDM-RII, RIIIDM-RIII. [file Data_Sheet_1.PDF]
